# Hyaluronic-Acid-Nanomedicine Hydrogel for Enhanced Treatment of Rheumatoid Arthritis by Mediating Macrophage–Synovial Fibroblast Cross-Talk

**DOI:** 10.34133/bmr.0046

**Published:** 2024-06-18

**Authors:** Yaping Wang, Jingrong Wang, Mengze Ma, Rui Gao, Yan Wu, Chuangnian Zhang, Pingsheng Huang, Weiwei Wang, Zujian Feng, Jianbo Gao

**Affiliations:** ^1^ Medical 3D Printing Center, The First Affiliated Hospital of Zhengzhou University, Zhengzhou 450000, China.; ^2^ Tianjin Key Laboratory of Biomaterial Research, Institute of Biomedical Engineering, Chinese Academy of Medical Sciences and Peking Union Medical College, Tianjin 300192, China.; ^3^ Key Laboratory of Innovative Cardiovascular Devices, Chinese Academy of Medical Sciences, Beijing 100144, China.

## Abstract

The occurrence of rheumatoid arthritis (RA) is highly correlated with progressive and irreversible damage of articular cartilage and continuous inflammatory response. Here, inspired by the unique structure of synovial lipid–hyaluronic acid (HA) complex, we developed supramolecular HA-nanomedicine hydrogels for RA treatment by mediating macrophage–synovial fibroblast cross-talk through locally sustained release of celastrol (CEL). Molecular dynamics simulation confirmed that HA conjugated with hydrophobic segments could interspersed into the CEL-loaded [poly(ε-caprolactone-*co*-1,4,8-trioxa[4.6]spiro-9-undecanone)–poly(ethylene glycol)–poly(ε-caprolaone-*co*-1,4,8-trioxa[4.6]spiro-9-undecanone] (PECT) nanoparticles to form the supramolecular nanomedicine hydrogel HA-poly(ε-caprolactone-*co*-1,4,8-trioxa[4.6]spiro-9-un-decanone)/PECT@CEL (HP@CEL), enabling fast hydrogel formation after injection and providing a 3-dimensional environment similar with synovial region. More importantly, the controlled release of CEL from HP@CEL inhibited the macrophage polarization toward the proinflammatory M1 phenotype and further suppressed the proliferation of synovial fibroblasts by regulating the Toll-like receptor pathway. In collagen-induced arthritis model in mice, HP@CEL hydrogel treatment substantial attenuated clinical symptoms and bone erosion and improved the extracellular matrix deposition and bone regeneration in ankle joint. Altogether, such a bioinspired injectable polymer-nanomedicine hydrogel represents an effective and promising strategy for suppressing RA progression through augmenting the cross-talk of macrophages and synovial fibroblast for regulation of chronic inflammation.

## Introduction

Rheumatoid arthritis (RA), which causes chronic pain and motor dysfunction, affects the quality of life of more than 20 million people worldwide and imposes a considerable financial burden on patients [1–3]. RA is characterized by chronic inflammation and persistent synovitis, leading to joint damage and involvement of heart, lung, and kidney organs [4,5]. Traditional surgery operations could cause large trauma and extensive adhesion and cannot be used repeatedly [6]. Although medications have been commonly used in clinic to improve inflammatory symptoms and delay disease progression including antirheumatic drugs and nonsteroidal anti-inflammatory drugs, glucocorticoids, adverse reactions such as severe infection and malignant tumor occurred because of frequent administration of high-dose drugs [7,8]. Therefore, novel therapeutic strategies for RA treatment are still urgently warranted.

The main cells in the synovial lining of the joint include macrophages and synovial fibroblasts. The 2 cells coordinate to play a role in maintaining the synovial fluid composition and joint integrity in a dynamic equilibrium state [9,10]. Under the pathological conditions of RA, macrophages and synovial fibroblasts are involved in the destruction of joints and pannus formation, leading to chronic inflammation [11,12]. Macrophages are typically differentiated into proinflammatory (M1) phenotype and anti-inflammatory (M2) phenotype, which both play crucial roles in pathophysiological immune responses [13]. In particular, M1 macrophages produce a range of proinflammation cytokines, such as tumor necrosis factor-α (TNF-α), interferon-γ, and interleukin-1β (IL-1β), resulting in excessive activation of Toll-like receptors (TLRs) [14]. Moreover, synovial fibroblasts in RA also act as a “proinflammatory” and “aggressive” phenotype, which could be activated by proinflammatory macrophages through activating TLR pathway [15]. Activated synovial fibroblasts produce inflammatory cytokine IL-6 and chemokine IL-8, as well as matrix metalloproteinases and other tissue destructive factors, which could, in turn, recruit macrophages and polarize them into M1 type in a positive feedback loop, leading to continued exacerbation of inflammation during the progression of RA [16]. However, the synergistic effect of synovial fibroblasts with macrophage function in a destructive inflammatory environment of RA has not been elucidated. Understanding the signals orchestrating macrophage and synovial fibroblasts may provide insights into the development of therapeutic strategies for RA.

Recently, hyaluronic acid (HA)-derived hydrogel with high water content and highly tunable mechanical properties received tremendous interests for RA treatment [17,18]. HA is the main component of synovial fluid and cartilage [19]. It has been widely reported that self-assembled layers or particles of phospholipids can bind with other molecules in the synovial fluid, particularly with HA, to form complexes that facilitate boundary lubrication, which are usually called lipid–hyaluronan complexes [20] or HA–lipid layers [21]. The unique structure of synovial lipid–HA complex could reduce the cartilage degradation and protect cells and anatomical structures against mechanical stresses. Therefore, HA-derived hydrogel showed potential in relieving osteoarthritis symptoms and improving joint function by reducing joint abrasion [22]. However, the design of a desired hydrogel for RA treatment is still challenging, where several properties are required: (a) injectable and fast in situ formation for direct intra-articular injection [23]; (b) high drug loading and locally sustained release of therapeutic agent to avoid systemic side effects; (c) extracellular matrix (ECM)-like porous microstructure for proliferation and infiltration of healing-related cells.

**Fig. 1.**
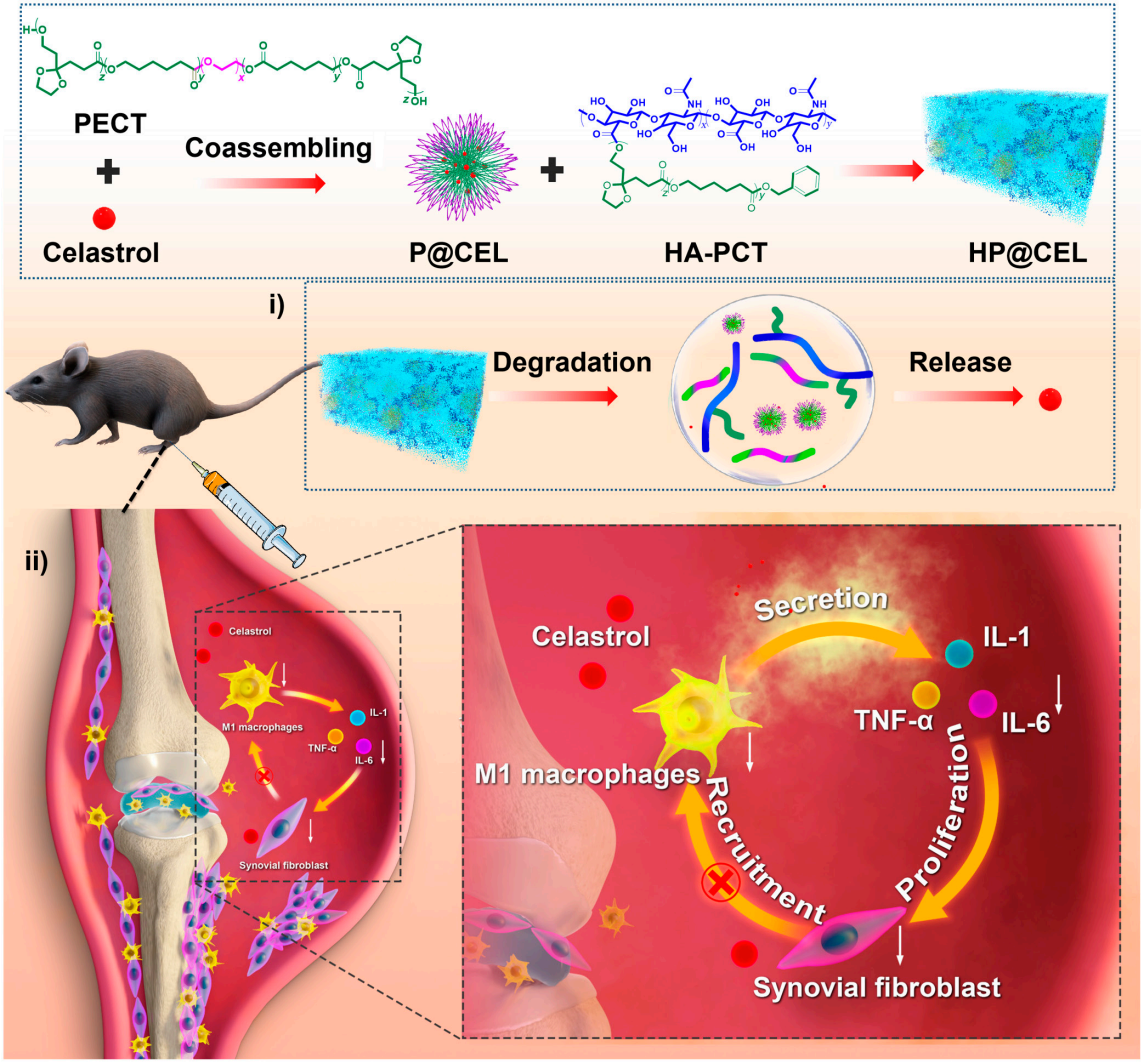
Diagram of the preparation of HP@CEL hydrogel for RA. Synovial lipid–HA complex-inspired HP@CEL hydrogel was coassembled by CEL-encapsulated PECT nanoparticles with HA-PCT polymers. (i) The degradation and sustained release of CEL from HP@CEL hydrogel. (ii) Mechanism of CEL regulating cross-talk of macrophages and synovial fibroblasts and HP@CEL that decreased M1 macrophage polarization and inhibited proliferation of synovial fibroblasts for relieving chronic inflammation during progression of RA.

Here, inspired by the structure of synovial lipid–HA complex, we fabricated a self-assembled HA-nanomedicine hydrogel to locally sustainable delivery of celastrol (CEL) for RA treatment through mediating the cross-talk of macrophage and synovial fibroblasts (Fig. 1). A hydrophobic polymer segment poly(ε-caprolactone-*co*-1,4,8-trioxa[4.6]spiro-9-un-decanone) (termed as PCT) was conjugated with HA backbone, which was expect to coassemble with amphiphilic [poly(ε-caprolactone-*co*-1,4,8-trioxa[4.6]spiro-9-undecanone)–poly(ethylene glycol) (PEG)–poly(ε-caprolaone-*co*-1,4,8-trioxa[4.6]spiro-9-undecanone] (termed as PECT) nanoparticles to replicate the structure of synovial lipid–HA complex and serve as lubricin to reduce the joint friction. In addition, combining with the thermosensitive characterization of PECT nanoparticles, the self-assemble behavior and polymer–nanoparticle interaction between HA-PCT and PECT nanoparticles endowed the hydrogel with well injectability and fast in situ forming capability. Moreover, as a novel drug delivery platform, PECT nanoparticle hydrogel has been proven to encapsulate drugs with high loading capability through hydrophobic interactions and achieve local and long-term drug release to increase the drug bioavailability and reduce the potential of drug-induced systemic toxicity [24]. In this work, CEL, a pentacyclic triterpenoid that belongs to the root of *Tripterygium wilfordii* [25–27], was loaded in PECT nanoparticles to target the TLR4/nuclear factor κB (NF-κB)/mitogen-activated protein kinase (MAPK) pathway and mediate the cross-talk of macrophage and synovial fibroblasts, aiming to mitigate the chronic inflammation and block the progression of RA. The therapeutic effects of HA-PCT/PECT@CEL (HP@CEL) hydrogel platform were fully investigated in a collagen-induced arthritis (CIA) mouse model. In addition, the potent therapeutic mechanism of harnessing macrophage polarization and mediating the cross-talk between macrophages and synovial fibroblasts as transmitted through TLR4/NF-κB/MAPK pathway was also clarified. It is demonstrated that bioinspired supramolecular HP@CEL hydrogel provides a remarkable therapeutic efficiency for RA.

## Materials and Methods

### Materials and animals

Sodium hyaluronate (molecular weight = 68 kDa; purity ≥ 95%) was purchased from Sigma-Aldrich (St. Louis, USA). 1,4,8-Trioxa[4.6]spiro-9-undecanone (TOSUO) and PECT were prepared according to the previous report [24,28]. Information for other chemical reagents, antibodies, and bacteria strains were provided in the Supplementary Materials. Female BALB/c (6 to 8 weeks) mice were purchased from Beijing Vital River Laboratory Animal Technology Co. Ltd. (Beijing, China).

### Public database analysis

The CEL target genes were clustered via the TCMSP database. Twenty-four drug targets were obtained with OB ≥ 17.84% and DL ≥ 0.78 as the screening conditions. UniProt, GeneCard, and OMIM databases were used to collect RA-related target genes and obtain potential target genes for CEL treatment of RA through Veeny 2.1 intersection. The protein–protein interaction (PPI) network diagram was constructed, and the name of the potential target gene was entered through the STRING database to select “*Homo sapiens*”. A visual network was established through Cytoscape software to reflect the complex relationship between CEL and RA-potential target genes. Besides, Kyoto Encyclopedia of Genes and Genomes (KEGG) pathway enrichment analysis of 24 CEL target genes was analyzed with Metascape database.

### Preparation of PECT@CEL nanoparticles

The full names of PECT, PECT@CEL (P@CEL), PCT, HA-PCT, and HP@CEL are listed in Table. P@CEL was prepared as follows: 100 mg of PECT and 5 mg of CEL were dissolved and mixed in tetrahydrofuran. Then, the above mixture was slowly added to 10 ml of double-distilled H_2_O. After stirring for 6 h, we centrifuged and removed the unwrapped CEL. The drug loading and encapsulation efficiency were determined by the ultraviolet-visible spectrophotometer and calculated according to the following formula.

$$\mathrm{CEL}\;\text{loading efficiency}\;\left(\%\right)=\frac{\text{Amount of}\;\mathrm{CEL}\;\text{in}\;\text{NPs}}{\text{Amount of}\;\text{NPs}}\times 100\%$$

$$\begin{array}{c} \mathrm{CEL}\;\text{encapsulation efficiency}\;\left(\%\right)\\ =\frac{\text{Actual loading}}{\text{Theoretical loading}\;\left(\text{dosage}\right)}\times 100\% \end{array}$$

**Table.** The list of abbreviations and full names of PECT, P@CEL, PCT, HA-PCT, and HP@CEL.

Abbreviation	Full name
PECT	Poly(ε-caprolactone-*co*-1,4,8-trioxa[4.6] spiro-9-undecanone)–poly(ethylene glycol)–poly(ε-caprolactone-*co*-1,4,8- trioxa[4.6]spiro-9-undecanone)
P@CEL	Celastrol-loaded poly(ε-caprolactone-*co*- 1,4,8-trioxa[4.6]spiro-9-undecanone)– poly(ethylene glycol)–poly(ε-caprolactone-*co*-1,4,8- trioxa[4.6]spiro-9-undecanone)
PCT	Poly(ε-caprolactone-*co*-1,4,8-trioxa[4.6] spiro-9-undecanone)
HA-PCT	Hyaluronic acid–graft–poly(ε-caprolactone- *co*-1,4,8-trioxa [4.6]spiro-9-undecanone)
HP@CEL	Hyaluronic acid–poly(ε-caprolactone-*co*- 1,4,8-trioxa[4.6]spiro-9-undecanone)@ celastrol-loaded poly(ε-caprolactone-*co*- 1,4,8-trioxa[4.6]spiro-9-undecanone)– poly(ethylene glycol)–poly(ε-caprolactone-*co*-1,4,8- trioxa[4.6]spiro-9-undecanone)

### Synthesis and characterization of HP@CEL hydrogel

HP@CEL hydrogel was prepared by coassembling between HA-PCT and CEL-loaded PECT nanoparticles. HP@CEL hydrogels were successfully prepared with different volume ratios of HA-PCT and HP@CEL solutions. Briefly, HA-PCT (5 wt%) and P@CEL (30 wt%) was dissolved and stored at temperature below 25 °C. The morphology of P@CEL nanoparticles released after hydrogel degradation was characterized by transmission electron microscopy (JEM-1011, JEOL, Tokyo, Japan).

### Molecular dynamics simulation of assembling behavior of PECT nanoparticles and HA-PCT

LAMMPS software was used to simulate the self-assembly behavior of coarse-grained PECT copolymers and HA-PCT in water environment, where yellow spheres represent coarse-grained PEG monomers and blue and red represent caprolactone (CL) and TOSUO monomers, respectively. The HA backbone is represented by green spheres. All simulations were repeated 3 times.

### Morphology and rheology

The freeze-dried samples of PECT, P@CEL, and HP@CEL hydrogels were observed and photographed under a scanning electron microscope (SEM). The rheology properties of hydrogels were evaluated by an AR 2000ex rheometer (TA Instruments).

### Polarization of macrophages in vitro

RAW264.7 was seeded in a 6-well plate at the density of 4 × 10^5^ cells per well and pretreated with lipopolysaccharide (LPS) (40 ng/ml) to obtain the M1-type macrophages. Then, phosphate-buffered saline (PBS), CEL, HA-PCT, PECT, or HP@CEL in fresh culture medium was added and incubated for further 48 h. Experimental details were described in the Supplementary Materials.

### Macrophage and synovial cell coculture

To study the effect of M1 proinflammatory factor on synovial fibroblasts, M1-type macrophages were cocultured with synovial fibroblasts. Experimental details were described in the Supplementary Materials.

### Micro-computed tomography analysis

Micro-computed tomography (micro-CT; nanoScan, Mediso) is used for image evaluation. The parameters of micro-CT were a voltage of 70-kVp, a current of 270 μA, and scanning time of 200 ms. At the end of scanning, 3-dimensional reconstruction was performed using RadiAnt DICOM Viewer and Mimics Research 19.0 software. Bone mineral density (BMD), trabecular thickness (Tb.Th), trabecular separation (Tb.Sp), trabecular number (Tb.N), and bone volume/total tissue volume (BV/TV) were measured and analyzed.

### Statistical analysis

All data were expressed as means ± SD. Statistical analysis was performed using GraphPad Prism 8.0 software. One-way analysis of variance (ANOVA) and Tukey’s multiple comparison test or Student’s *t* test were used to evaluate the differences between groups. Statistical significance was expressed as *P < 0.05, **P < 0.01, and ***P < 0.001.

## Results

### Preparation and characterization of hydrogel (PECT, P@CEL, and HP@CEL)

**Fig. 2.**
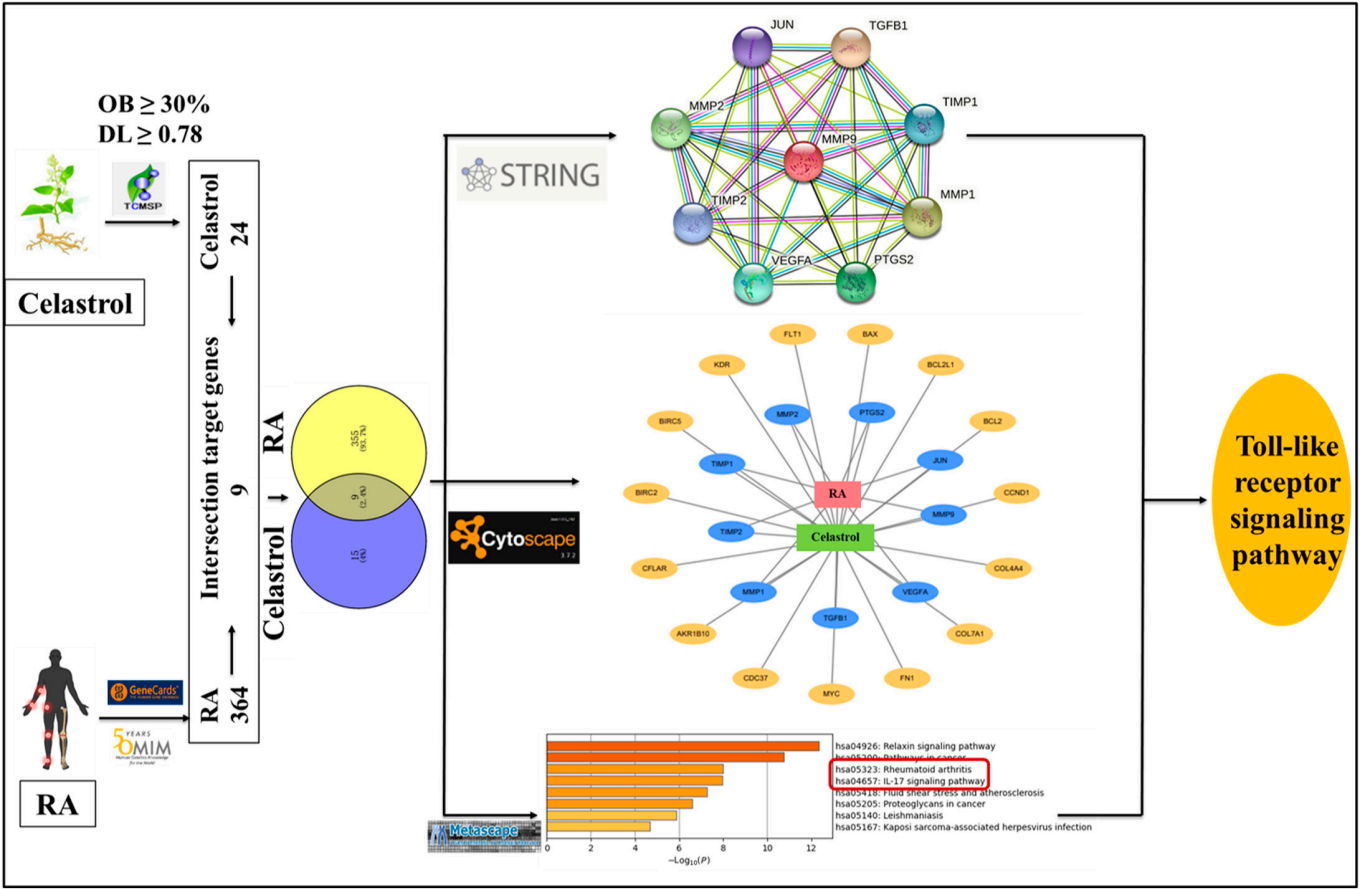
The workflow of TLR signaling pathway prediction in RA.

To reveal the potential targets of CEL in the treatment of RA, we searched and collected 24 potential target genes belonging to CEL from TCMSP based on OB ≥ 17.84% and DL ≥ 0.78 as the screening conditions (Table S1). After searching the GeneCard and OMIM databases, repeated target genes were excluded. A total of 364 target genes involved in RA were obtained. Similarly, 24 target genes involved in CEL. Using Venny 2.1 software analysis, 9 potential targets for the intersection of RA and CEL genes were obtained (Table S2). Sequentially, the 9 target genes were imported into the STRING database to obtain the PPI network map (Fig. 2 and Fig. S1). As seen in the CEL–RA-potential target gene network (Fig. 2 and Fig. S2), many target genes can be regulated by multiple compounds in RA, suggesting that CEL for RA therapy has multicomponent and multitargeted biological attributes. Besides, KEGG pathway enrichment analysis of 24 potential target genes of CEL was performed via Metascape database. The results indicated that these potential target genes were highly involved in RA and IL-17 signaling pathway (Fig. 2 and Fig. S3). On the basis of the results of the above comprehensive analysis, it can be concluded that CEL could affect the target of RA through regulating the TLR pathway.

In healthy joints, there is full of lubricant synovial fluid, which composed of HA, lubricin, and surface-active phospholipids [29]. We further modified the HA with hydrophobic segment PCT to coassemble with PECT nanoparticles to biomimetic the synovial lipid–HA complex. The PECT nanoparticle hydrogel was previously reported by our laboratory with advantages including injectability, degradable and biocompatibility, and controlled and sustainable drug delivery [24], holding great potential as local drug delivery systems. Therefore, the CEL could be encapsulated in the HA-PCT–PECT hydrogel, which was expected to locally sustain the release and exert a long-lasting effect in inflammation regulation.

**Fig. 3.**
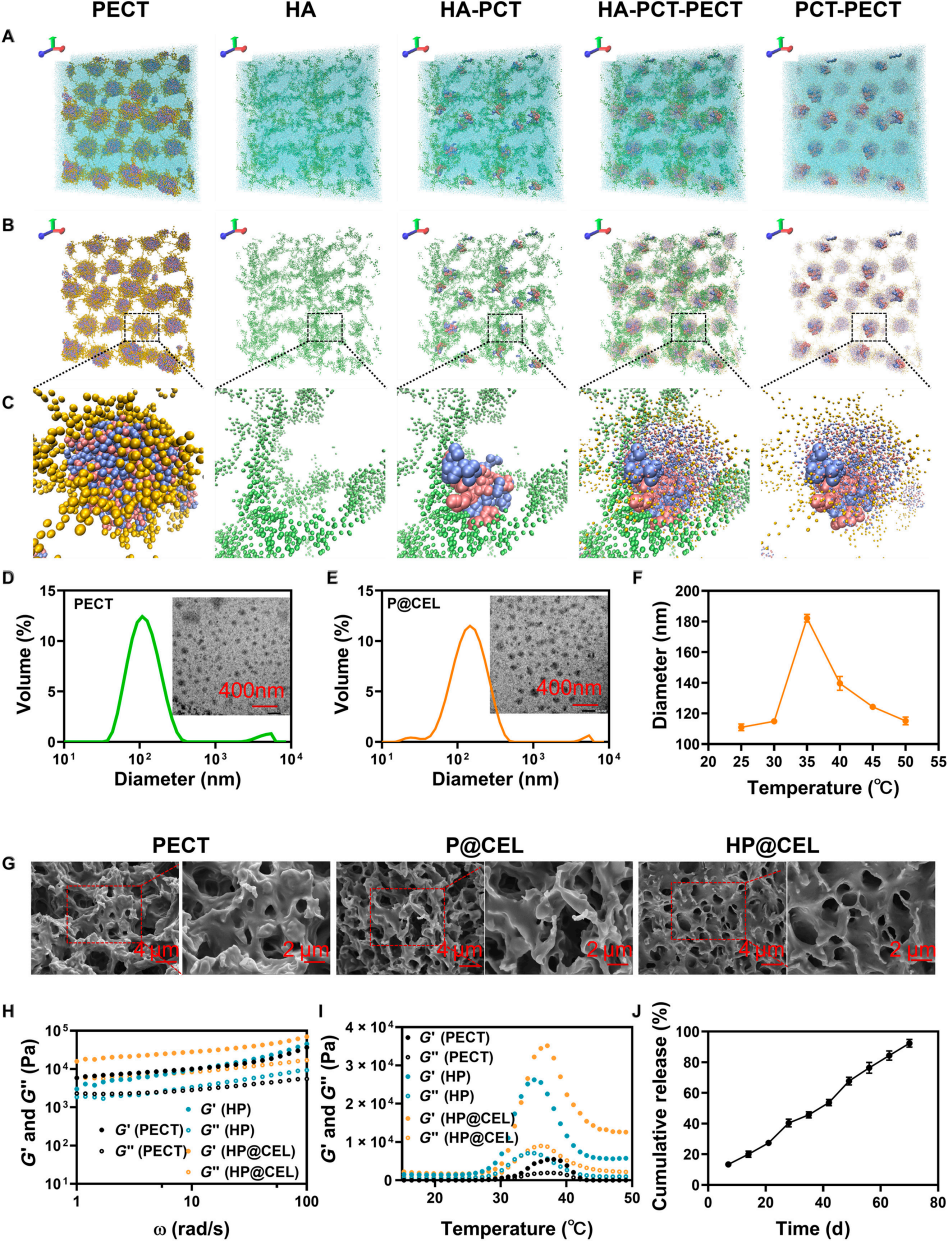
Molecular dynamics simulation of PECT and HP hydrogel. After HA-PCT was coassembled with PECT nanoparticles, the hydrophobic side chains interspersed inside the PECT nanoparticles. (A and B) HA-PCT interspersed into PECT micelles. (C) Local atom distribution of one PECT micelle with interspersing HA-PCT side chain. (D and E) Size distribution and transmission electron microscopy images of PECT and P@CEL nanoparticles in aqueous solution at the concentration of 2 mg/ml. (F) Sizes of PECT nanoparticles incubated under different temperatures at the concentration of 2 mg/ml. (G) Representative SEM images of PECT, P@CEL, and HP@CEL hydrogels. (H and I) Rheological analysis of PECT, HP, and HP@CEL hydrogels as a function of angular frequency (H) and shear strain (I), respectively. (J) The cumulative release profile of CEL from the hydrogel in vitro.

First, PECT and PCT were prepared by ring-opening polymerization according to the previous method (Figs. S4 to S6) [28]. Then, HA conjugated with PCT was obtained through typical esterification reaction of carboxyl and hydroxyl. Results of ^1^H nuclear magnetic resonance and gel permeation chromatography (Figs. S7 to S9) showed successful synthesis of PECT and PCT. The ratio of PEG, CL, and TOSUO and the actual monomer ratio calculated by nuclear magnetic resonance proved the successful synthesis of PECT (Tables S3 and S4). The substitution degree of PCT in HA-PCT was 13.14 % (Fig. S10). The peak at 2,945.3 cm^−1^ was attributed to the methylene group of PCT, the hydroxyl peak at 3,400 cm^−1^ was attributed to the hydroxyl peak of HA and HA-PCT, while PCT did not contain this peak, indicating that HA-PCT was successfully synthesized (Fig. S11). The P@CEL nanoparticles aqueous solution was prepared by nanoprecipitation method. Then, HP@CEL hydrogel was obtained through simply mixing the P@CEL nanoparticles and HA-PCT aqueous solution. First, to reveal the assemble mechanism of HA-PCT and PECT nanoparticles, we carried out molecular dynamic simulation and studied the formation process of nanoparticles with full periodic boundary condition. We used LAMMPS software to simulate the self-assembly behavior of coarse-grained PECT copolymers in water environment, where yellow spheres represent coarse-grained PEG monomers and blue and red represent CL and TOSUO monomers, respectively (Fig. S12). The structural distribution of PECT polymers shows that CL and TOSUO polymers with hydrophobic structures tend to enrich the interior of the aggregates, while the hydrophilic group PEG tends to accumulate outside the nanoparticles. The random copolymer can spontaneously aggregate to the morphology of ordered nanoparticles in an aqueous environment, which verifies the successful preparation of our PECT nanoparticles in molecular theory. A high-resolution molecular dynamics simulation was established with a hydrophobic side-chain HA structure in an explicit water environment to study the insertion of hydrophobic chains into the hydrophobic part of the micelle caused by hydrophobic forces in the presence of PECT micelles. The PECT copolymer was generated through random walk and randomly displaced into the simulation box. Different types of PECT monomer, which are PEG, CL, and TOSUO, are represented by coarse-grained atom showing in different colors. The empty space of the simulation box was filled with coarse-grained water atom; each atom represents 4 water molecules. To obtain stable PECT solution, the morphology of polymer chain went through a steepest descent gradient energy minimization method. The system was then equilibrated using constant number, pressure, and temperature (NPT ensemble) for 2.5 μs until the spherical morphology of PECT was obtained (Fig. S12). HA-PCT side chain was also generated through the same approach as PECT. After energy minimization, HA-PCT with PECT micelle was equilibrated using NPT ensemble for 5 μs until it reached an equilibrium state as shown in Fig. 3 (A to C). The simulation box was expanded once in *x* and *z* directions for better visualization, showing in Fig. 3B and C. Radius of the PECT bead is 0.3. To visually observe the distribution of the hydrophobic side-chain PCT and PECT components, we set the radius of the PECT bead to 0.1, the radius of the hydrophilic main-chain HA bead to 0.2, and the radius of the hydrophobic side-chain PCT to 0.6. Through the local amplification map (Fig. 3C), we observed that the hydrophobic side chain of HA-PCT interspersed into the hydrophobic part of PECT nanoparticles, while HA was mainly wrapped in the hydrophilic layer of PECT nanoparticles, demonstrating a synovial lipid–HA complex-like structure that benefits in reducing joint friction through in situ injection.

As shown in Fig. 3D and E and Table S5, P@CEL showed uniform with size of ~146 nm and a narrow size distribution [polymer dispersity index (PDI) = 0.24]. In addition, compared with PECT nanoparticles, size of P@CEL nanoparticles was slightly increased, suggesting that the encapsulated CEL had little effect on assembly behavior of the nanoparticles. The assembly of nanoparticles in HP@CEL hydrogel degradation solution remained intact, which proved that the gelation process did not destroy the morphology of drug-loaded nanoparticles (Fig. S13). Sol-to-gel transition of HP hydrogel was examined by the test tube inverting method. Results showed that after mixing, the solutions of HA-PCT and P@CEL with a concentration of 5 and 30 wt% exhibited typical thermosensitive properties, which was initially in a sol state at 25 °C and formed immediately after incubation at 37 °C (Fig. S14). Therefore, unless otherwise specified, HA-PCT and P@CEL with a concentration of 5 and 30 wt% were used in the following experiments. As shown in Fig. 3F, the size of nanoparticles continued to increase as the temperature increased from 25 to 35 °C, which is possibly due to the micellar aggregation, suggesting a micellar aggregation mechanism for the gelation formation at high temperature. SEM images (Fig. 3G) of lyophilized samples of PECT, P@CEL, and HP@CEL hydrogels showed an interconnected porous network structure. In addition, the injectability and temperature sensitivity of HP@CEL hydrogels were verified by rheological tests. The storage modulus (*G*′) of HP@CEL hydrogel was higher than the loss modulus (*G*″) at 37 °C with the change of angular frequency, showing a solid-like hydrogel state (Fig. 3H). Figure 3I showed that PECT, HP, and HP@CEL hydrogels were all showed typical thermosensitive properties, which the modulus remarkably increased with the temperature increased to about 35 °C. It was worth noting that HP@CEL hydrogel showed the highest modulus under physiological conditions (37 °C), suggesting the stable and fast-forming capability after in situ injection. In addition, HP@CEL hydrogel can be easily injected through a 25-gauge syringe (Fig. S15), also reflecting the injectability of HP@CEL hydrogel. The mechanism of thermosensitivity of HP hydrogel was investigated by dynamic light scattering under different temperatures. In vitro degradation (Fig. S16) indicated that HP@CEL hydrogels with or without the presence of hyaluronidase both displayed a sustainable degradation behavior. In addition, the hyaluronidase could accelerate the degradation of the hydrogel, of which 15.3% of hydrogel degraded during 18 d in vitro, owing to the fact that the enzymolysis of HA reduced the cross-linking network. A long degradation cycle was beneficial for prolonging the function of the hydrogel through long-term drug delivery. Subsequently, in vitro release (Fig. 3J and Fig. S17) indicated that CEL could be continuously released from HP@CEL hydrogel. The accumulative release for CEL was about 92% after 70 d. Furthermore, the release behavior of CEL from P@CEL and HP@CEL in the presence of hyaluronidase was also studied. We found that both P@CEL and HP@CEL showed a sustainable CEL release behavior in vitro in the presence of hyaluronidase. In addition, drug release of HP@CEL was slower than that of P@CEL, indicating that HA with hydrophobic side chains of PCT could enhance the mechanical properties and stability of PECT, thus conducive for prolonging the period of drug release. This was beneficial for improving the bioavailability of drugs that avoid the side effects and unnecessary infection caused by repeated administration in joint cavity.

### Cell compatibility and differentiation in vitro

**Fig. 4.**
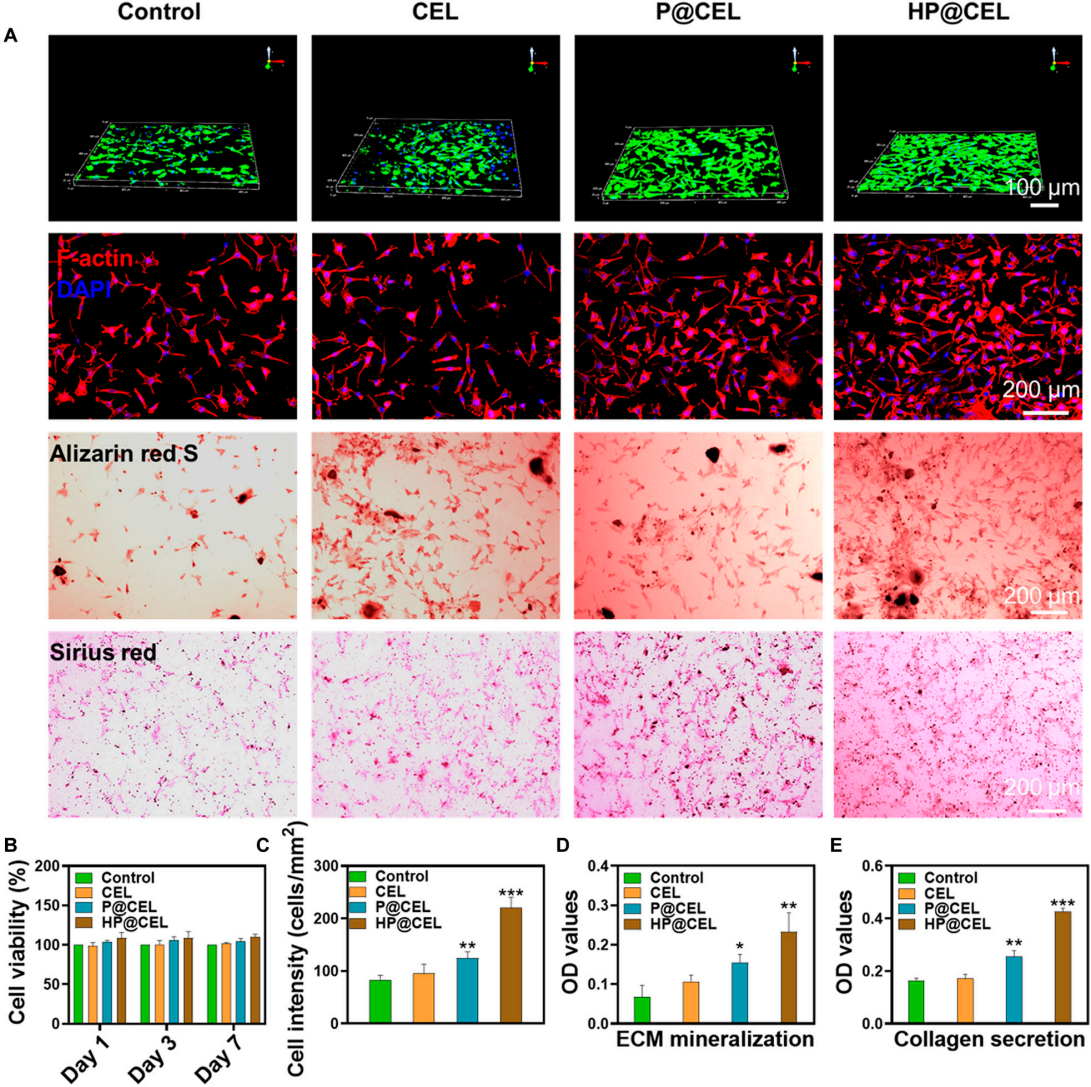
Cell proliferation and differentiation of BMSCs after HP@CEL treatment. (A) The 3-dimensional construction of confocal laser scanning microscopy (CLSM) images of BMSCs on the hydrogel after incubation for 3 d: cell proliferation, Alizarin red S, and sirius red staining. (B) Cell viability Cell Counting Kit-8 (CCK8) assay. (C) The cell morphology and proliferation of BMSCs after different treatments. (D and E) Semiquantitative analysis of Alizarin red and sirius red staining. *P < 0.05, **P < 0.01, and ***P < 0.001, between the indicated groups. DAPI, 4′,6-diamidino-2-phenylindole; OD, optical density.

Next, we studied the effect of HP@CEL hydrogel on the viability and proliferation of bone marrow mesenchymal stem cells (BMSCs). After 3 d of culture, compared with the control group, the substantial proliferation of BMSCs was observed in HP@CEL hydrogel groups, suggesting that HP@CEL hydrogel with 3-dimensional porous microstructure was beneficial for nutrients supplying and proliferation of cells (Fig. 4A). In addition, it was reported that HA as the main component of synovial fluid and cartilage ECM could also attribute to the proliferation of chondrocyte. The cell viability of BMSCs on the days 1, 3, and 7 was about 108.4%, 109.1%, and 110.1%, respectively, indicating that BMSCs maintained a high cell viability during culture in HP@CEL hydrogel (Fig. 4B). Representative images of BMSCs (Fig. 4A and C) demonstrated that the BMSCs cultured with HP@CEL hydrogel exhibited higher cell density compared with other treatment and further certificated that HP@CEL hydrogel may facilitate the proliferation of BMSCs. Then, calcium nodules were stained with Alizarin red S to explore the ECM mineralization of BMSCs. As shown in Fig. 4A and D, ECM mineralization induced by P@CEL and HP@CEL was about 2.1- and 3.3-fold than that in control group, respectively. As shown in Fig. 4A and E, compared with the control group and the CEL group, the collagen secretion determined by sirius red staining of BMSCs treated with P@CEL or HP@CEL were significantly increased. These colorimetric analyses demonstrated that coincubation with the hydrogel facilitated the proliferation and differentiation of BMSCs, which may be attributed to the HA components and ECM-like 3-dimensional microstructure of the hydrogel [30,31].

### Anti-inflammation efficacy of HP@CEL hydrogel and the macrophage–synovial fibroblast cross-talk in vitro

**Fig. 5.**
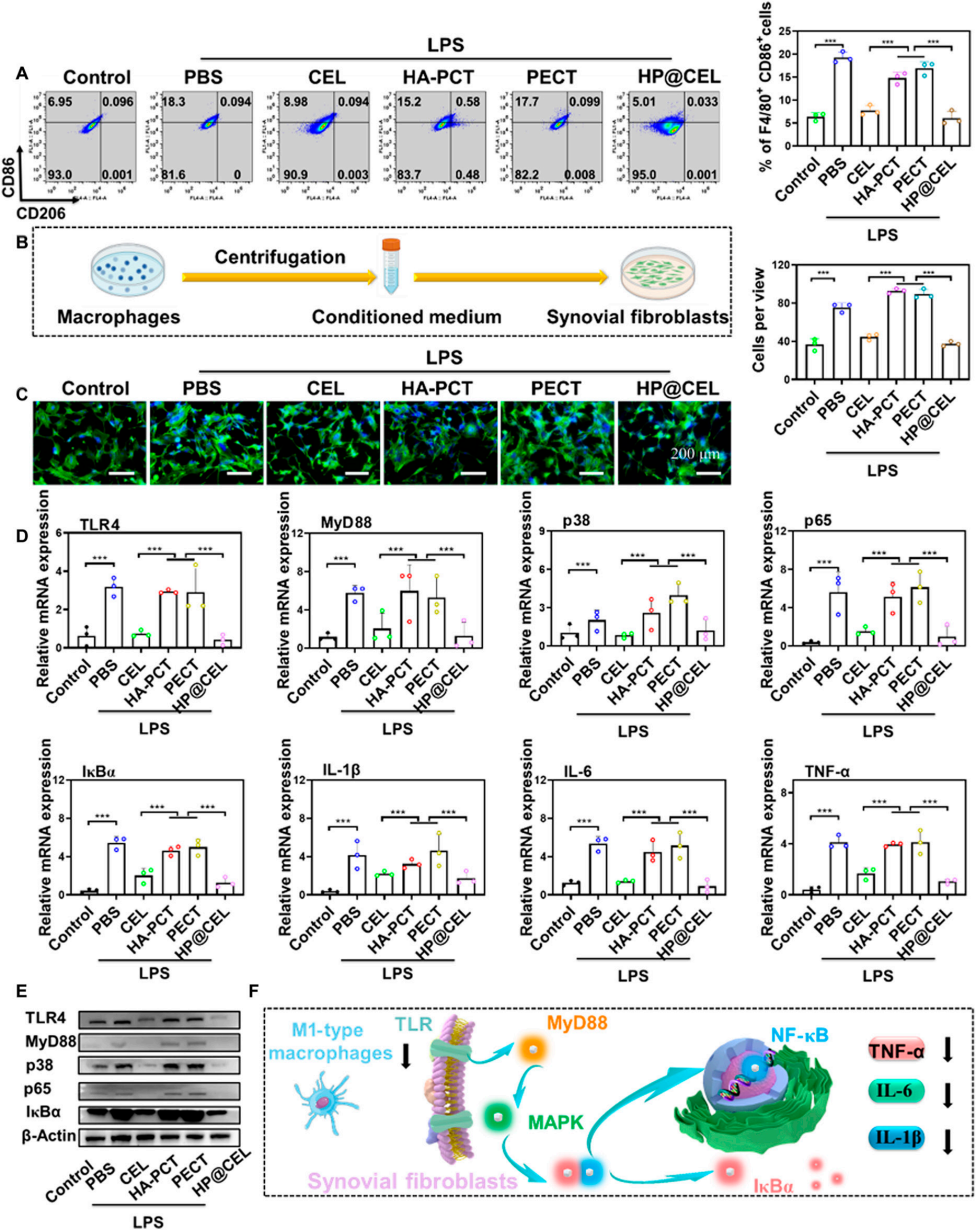
HP@CEL hydrogel regulate macrophage polarization and the macrophage–synovial fibroblast cross-talk. (A) Flow cytometry analysis of CD86 expression and the percentage of M1-type (F4/80^+^CD86^+^) macrophages after receiving different treatments (*n* = 3). (B) Schematic illustration of the macrophage–synovial fibroblast cross-talk. (C) Typical CLSM images and the statistical number of synovial fibroblasts incubated with supernatant derived from M1 macrophages after receiving different treatments for 24 h in vitro. (D) The mRNA expression levels of TLR4, MyD88, p38, p65, IκBα, as well as IL-1β, IL-6, and TNF-α detected by RT-qPCR (*n* = 3). (E) Protein expression level of TLR4, MyD88, p38, p65, and IκBα in synovial fibroblasts determined by Western blot. (F) The signal pathway of TLR activation mediates cross-talk of macrophages and synovial fibroblasts. ***P < 0.001 between the indicated groups.

The functional polarization of macrophages plays an important role in the regulation of inflammation and the repair of damaged tissues [32]. Studies have shown that CEL can inhibit macrophage transcription factor NF-κB signaling pathway in the treatment of arthritis animal models [27]. Subsequently, the response of RAW264.7 to different treatments was investigated. First, M1-type macrophages were obtained through pretreated and incubated of LPS for 48 h. Cells without pretreatment were set as negative control. Results in Fig. 5A showed that after gating on F4/80^+^ and side scatter–area (SSC-A) (Fig. S18), the percentage of CD86-positive macrophages (M1 phonotype) treated by CEL and HP@CEL was 8.98% and 5.01%, respectively, which were significantly lower than that in PBS (18.3%), HA-PCT (15.2%), and PECT (17.7%), suggesting that the HP@CEL hydrogel could reduce the inflammatory response of M1-type macrophages. The inappropriate activation of TLRs in the inflammatory microenvironment would induce abnormal proliferation of synovial fibroblasts to amplify and sustain the inflammatory response [33]. Then, the bone immunomodulatory effect of M1 macrophage polarization on synovial fibroblasts was further detected. The supernatants of M1 macrophages cocultured with PBS, CEL, HA-PCT, PECT, and HP@CEL were collected to stimulate synovial fibroblasts for 24 h (Fig. 5B). As shown in Fig. 5C, CEL and HP@CEL can remarkably down-regulate the activation and proliferation of synovial fibroblasts. Reverse transcription quantitative polymerase chain reaction (RT-qPCR) was used to analyze the expression of osteogenesis-related protein-coding genes in synovial fibroblasts after treatment, and detailed sequences of primers used in RT-qPCR were given in Table S6. As shown in Fig. 5D, compared with synovial fibroblasts treated by supernatants of M1 macrophages cocultured with PBS, HA-PCT, and PECT hydrogels, the levels of TLR4, myeloid differentiation factor 88 (MyD88), p38, p65, inhibitor of NF-κBα (IκBα), as well as IL-1β, IL-6, and TNF-α in synovial fibroblasts treated by supernatants of M1 macrophages cocultured with CEL and HP@CEL were significantly reduced. Then, the intracellular signal transduction proteins after TLR activation were detected by Western blot to explore the mechanism of HP@CEL-mediated apoptosis of synovial fibroblasts. As shown in Fig. 5E, the expression level of TLR4, MyD88, p38, p65, and IκBα of synovial fibroblasts was significantly decreased after treating with HP@CEL in comparison with the LPS. These results demonstrated that the HP@CEL could effectively mediate the cross-talk of macrophages and synovial fibroblasts through inhibiting the M1 phonotype macrophages and the proliferation of synovial fibroblasts by down-regulating TLR4/NF-κB signaling pathway, leading to a lower level of proinflammatory cytokines, which hold potential for relieving the chronic inflammation and blocking the progression of RA. The cross-talk between macrophages and synovial fibroblasts mediated by TLR-activated signaling pathways is shown in Fig. 5F and Fig. S19.

### In vivo therapeutic effect in CIA animal model by HP@CEL

**Fig. 6.**
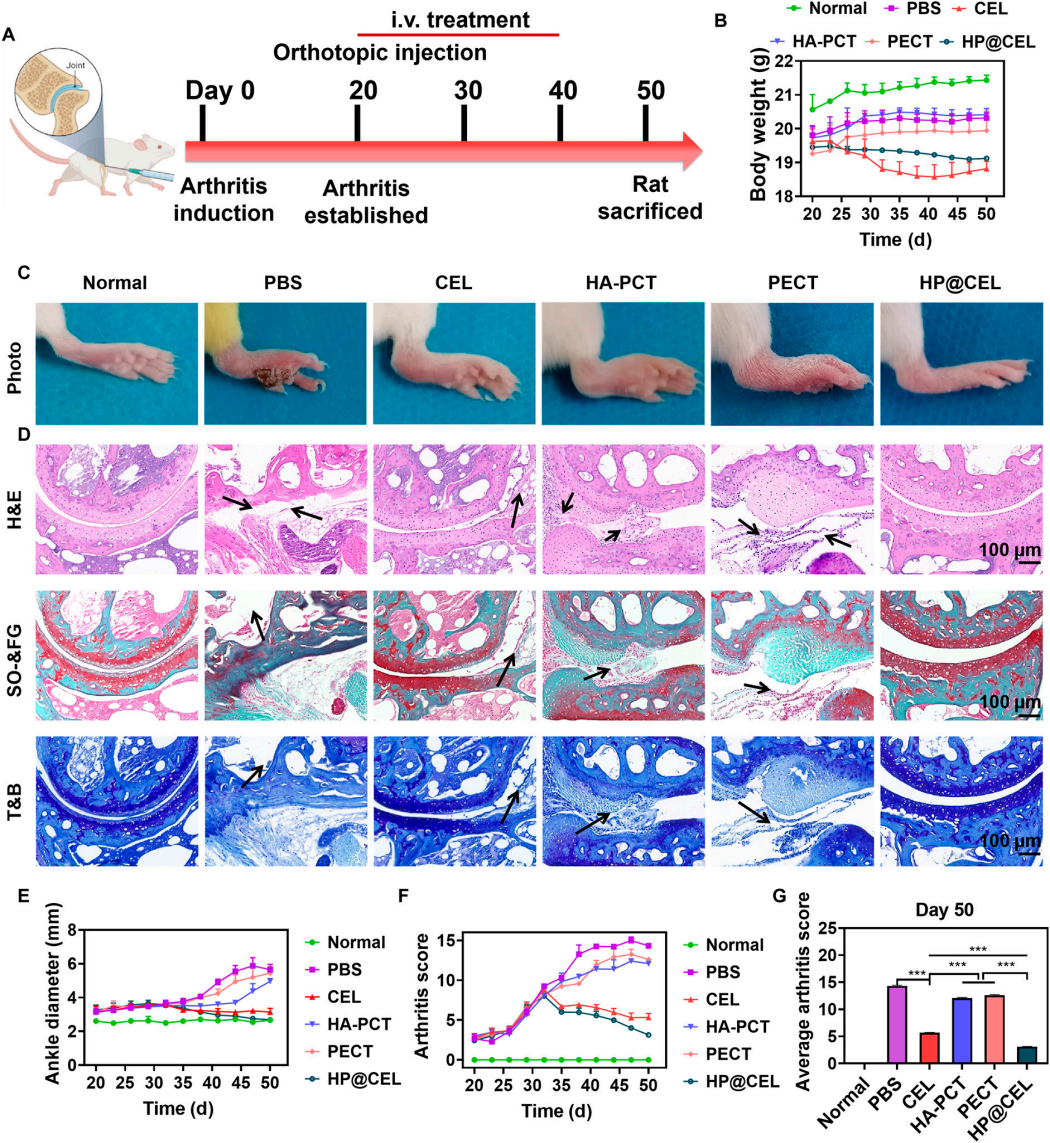
Therapeutic efficacy of HP@CEL hydrogel with RA in vivo. (A) Process diagram of HP@CEL hydrogel in the treatment of advanced arthritis. (B) The body weight of the mice was evaluated every 3 d after receiving different treatments. (*n* = 3). (C) Representative gross lesions after receiving different treatments at 50 d. (D) Pathological staining with H&E, Safranin O-Fast green (SO-FG) and Toluidine Blue (T&B). CIA clinical symptoms are effectively attenuated by injection of HP@CEL hydrogel in situ (the black arrows indicated bone erosion and cartilage destruction), including (E) ankle diameter (in millimeters) of the paw of mice (*n* = 3), (F) clinical scores every 3 d (*n* = 3), and (G) average arthritis clinical scores at 50 d. Data were shown as means ± SDs. ***P < 0.001 between the indicated groups. i.v., intravenous.

To evaluate its degradation behavior and biocompatibility in vivo, we subcutaneously injected HP@CEL hydrogel into the back of mice. The results showed that HP@CEL gradually degraded in vivo over time and was completely absorbed on the 50th day after injection (Fig. S20A). Hematoxylin and eosin (H&E) staining (Fig. S20B) at 1, 7, 14, and 21 d after injection showed that there was little inflammatory cell infiltration at the injection site and there was no obvious pathological phenomenon in the heart, liver, spleen, lung, and kidney, which verified the biosafety of HP@CEL hydrogel for in vivo application. Subsequently, the therapeutic effect of HP@CEL treatment was evaluated in the CIA model. After 20 d of arthritis induction, the ankles and soles of the mice showed severe swelling. Saline was injected at intra-articular as control, and CEL at a dose of 10 mg/kg was injected intravenously every 10 d. HP@CEL hydrogel (50 μl with CEL loading content of 0.6 wt%) was injected in situ into the intra-articular of the mice (Fig. 6A). As shown in Fig. 6B, compared with the normal mice, the body weight of the mice in all experimental groups decreased to varying degrees, indicating that RA seriously affected the normal behavior of the animals. Moreover, more weight loss was observed in the CEL treatment group, which might attribute to the side effects of intravenous administration of CEL. As shown in Fig. 6C and E, HA-PCT and PECT without CEL had relatively low effects on the reduction of paw thickness and ankle diameter in arthritis mice. In contrast, compared with CEL treatment, HP@CEL treatment showed higher efficacy in reducing ankle and paw swelling, and its ankle diameter and paw thickness were closer to the normal group at the end point. Further histopathological analysis of joint tissue was performed to evaluate the therapeutic effect of HP@CEL. As shown in Fig. 6D, the H&E staining tissue of the saline group showed severe synovial hyperplasia, accompanied by bone and cartilage destruction, indicating that it is difficult to repair without intervention in RA. The HA-PCT and PECT groups displayed a moderate effect in reducing these symptoms, indicating that the hydrogel in situ injection could provide physical support and as a lubricant to improving the progression of RA. While free CEL could reduce synovial inflammation and decrease the loss of cartilage to some extent, therapeutic effect was limited by the short half-life after intravenous injection. Notably, the HP@CEL treatment showed a remarkable reduction in bone erosion and mild synovial hyperplasia, probably mainly attributing to the locally sustainable drug release and immunomodulatory effect of HP@CEL. Safranin O/fast green staining and toluidine blue staining showed that the knee cartilage of mice in the PBS group was significantly damaged and severe cartilage damage was also observed in the articular cartilage of mice in the HA-PCT and PECT groups, while the knee cartilage of mice in the HP@CEL group was effectively repaired. Results in Fig. 6C demonstrated that HP@CEL treatment induced the most matrix deposition and improved levels of cartilage regeneration, which were closer to those of the normal articular tissue. Results in Fig. 6F and G also showed that the clinical arthritis score was significantly degreased after HP@CEL treatment, indicating a good therapeutic effect on advanced arthritis. These results indicated that HP@CEL effectively alleviated synovial inflammation and reduced cartilage destruction in mice with advanced arthritis.

**Fig. 7.**
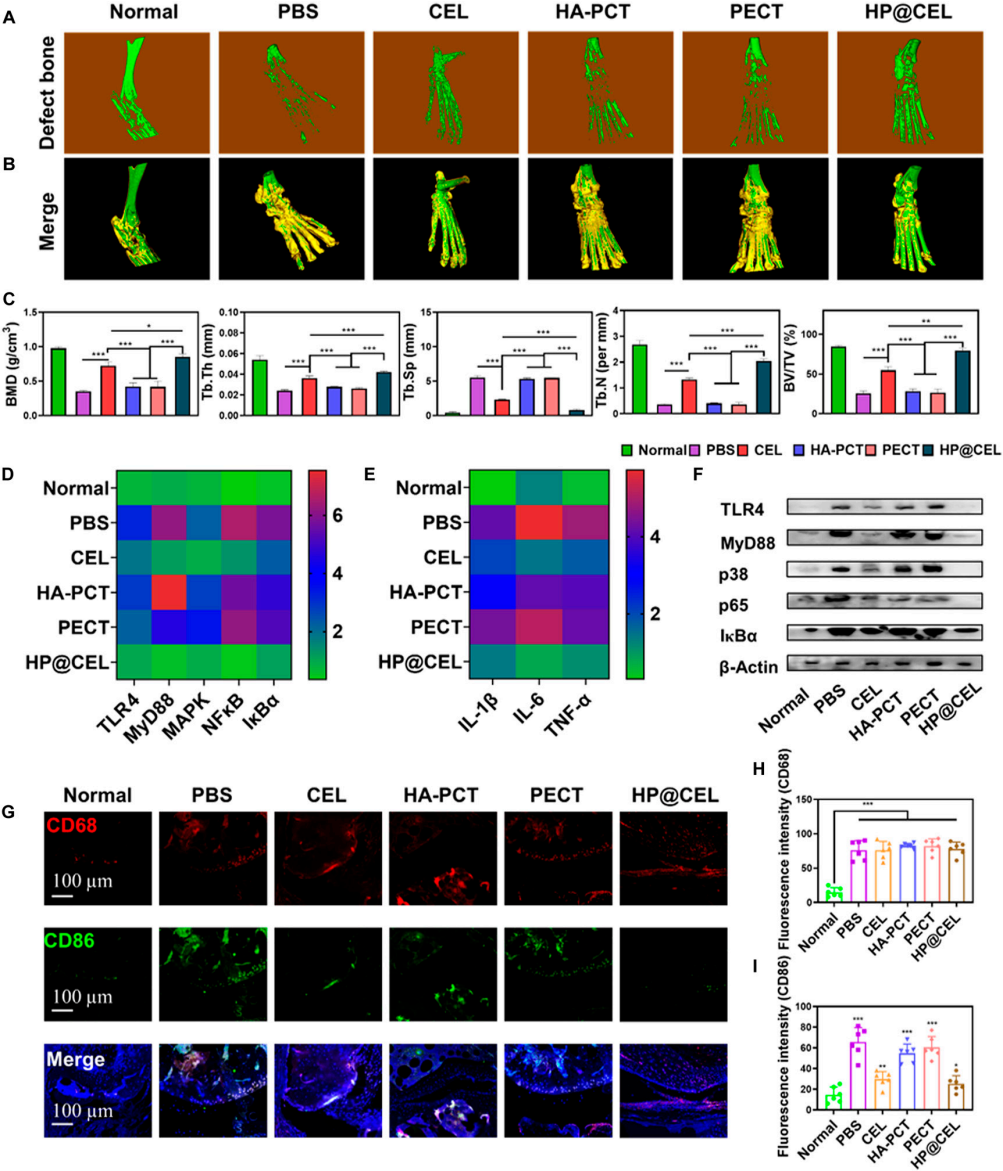
HP@CEL hydrogel reversed bone erosion with RA. (A) Micro-CT images of bone erosion. (B) Representative micro-CT images of the ankle joint at the end of the experiment in different treatment groups. (C) BMD, Tb.Th, Tb.Sp, Tb.N, and BV/TV. (D and E) The mRNA expression levels of TLR4, MyD88, p38, p65, IκBα, and proinflammatory cytokines IL-1β, IL-6, and TNF-α were detected by RT-qPCR (*n* = 3). (F) Protein expression level of TLR4, MyD88, p38, p65, and IκBα in joint tissue determined by Western blot. (G) Immunofluorescence images of representative macrophage surface markers. (H and I) fluorescence intensity statistics. The data were expressed as means ± SD (*n* = 3 independent animals). Two-sided Student’s *t* test was used for statistical significance. *P < 0.05, **P < 0.01, and ***P < 0.001, between the indicated groups.

The erosion and destruction of articular cartilage is one of the key causes of disability and poor prognosis in RA [34]. The therapeutic effect of HP@CEL on inhibiting bone erosion in arthritis model was confirmed by 3-dimensional bone micro-CT. As shown in Fig. 7A and B, on the 30th day after treatment, the inflammatory ankle joint in the saline group showed rough bone surface and severe bone erosion, and BMD was significantly lower than that in the normal group (Fig. S21 and Movies S1 to S6). Furthermore, CEL showed moderate efficacy in reducing bone erosion. Notably, the HP@CEL group showed a smooth bone surface and higher BMD in the ankle joint, which was almost similar to the normal group (Fig. 7C). In addition, HP@CEL treatment also showed the effectiveness of improving defect bone parameters. The treatment group showed a significant increase in the number of Tb.N, Tb.Th, and BV/TV, as well as an effective decrease in Tb.Sp, indicating that HP@CEL treatment can effectively terminate the progression of RA bone destruction while repairing bone erosion.

### HP@CEL alleviated the chronic inflammation and harnessed the macrophage polarization in RA

To understand the underling mechanism of the therapeutic effect of HP@CEL treatment, the expression of specific genes in articular tissue was quantitatively determined. As shown in Fig. 7D, E, in control group, mRNA expression level of proinflammatory cytokines including IL-1β, IL-6, and TNF-α was highly up-regulated compared with the normal group, demonstrating the persistent and high level of inflammation in RA. In comparison with other treatment, HP@CEL treatment conspicuously down-regulated mRNA expression levels of these proinflammatory cytokines, suggesting its superiority in anti-inflammatory effect in vivo. Furthermore, the expression of signal-transduction-associated proteins was detected by RT-qPCR and Western blot. The mRNA expression level of TLR4, MyD88, MAPK, NF-κB, and IκBα was down-regulated after treated with HP@CEL. In addition, the protein expression level of TLR4, MyD88, p38, p65, and IκBα in CEL and HP@CEL-treated groups was significantly down-regulated in comparison with other treatments (Fig. 7F), suggesting that the anti-inflammatory effect was mainly attributed to locally sustain the release of CEL that inhibited the excessive activation of TLR4 in the chronic inflammatory microenvironment and further block the downstream of NF-κB/MAPK pathway.

The healing process of RA is strongly influenced by the polarity of macrophages. Reducing proinflammatory M1 macrophages also commits to reduce the levels of inflammation-related cytokines and inhibits the positive feedback loop of chronic inflammation formed between macrophages and synovial fibroblast, thereby preventing cartilage damage and bone resorption in RA. Therefore, CD68 is used as the biomarker for all subsets of macrophages, while CD86 is used to label M1-type macrophages to identify the macrophage infiltration and differentiation in the articular tissue. As shown in Fig. 7G, compared with normal group, the populations of macrophages in other groups were all elevated to varying degrees, indicating the important role of macrophage infiltration of the inflammatory response during the progression of RA. Results in Fig. 7H and I showed that the distribution of M1 macrophages was higher in the PBS group (65.5%) of the articular tissue, while significantly down-regulating after treated by HP@CEL (25.2%), suggesting that the HP@CEL could regulate the macrophage polarization to block the chronic inflammatory of RA. Together, these above results demonstrated that HP@CEL displayed immunomodulatory effect including down-regulating the proinflammatory cytokines, harnessing the macrophage polarization, and mediating the macrophage and synovial fibroblast cross-talk transmitted through TLR4/MAPK/NF-κB signal pathway, thus effectively intervening the positive feedback of the chronic inflammation and improving the osteogenesis in RA.

### Microenvironmental changes in HP@CEL-treated RA

**Fig. 8.**
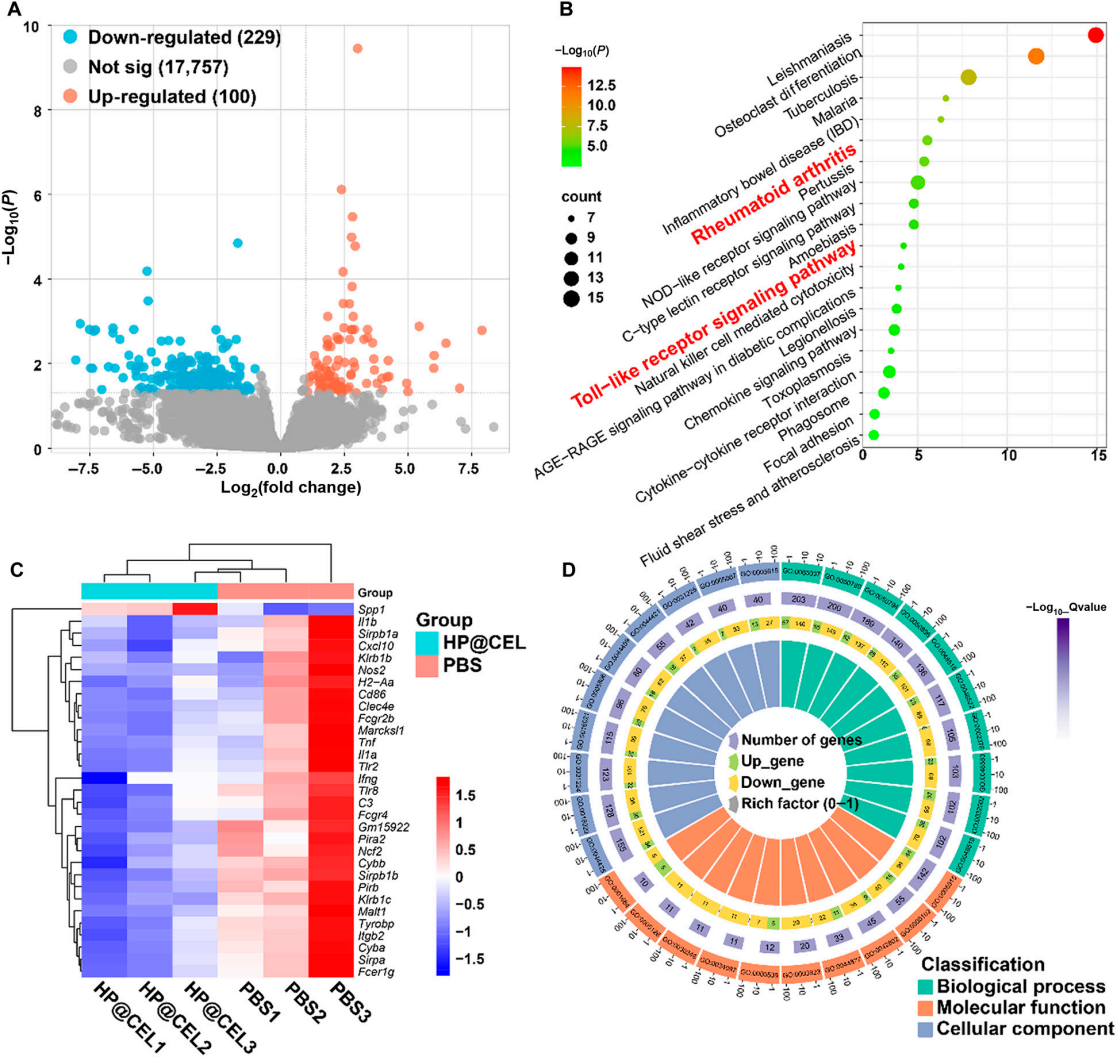
Gene expression and bioinformatics analysis across the HP@CEL. (A) Volcano plot of the differentially expressed genes between groups. (≥2-fold difference; red, up-regulated genes; blue, down-regulated genes). (B) The gene enrichment KEGG pathway analysis. In general, HP@CEL can effectively regulate the inflammatory microenvironment, and TLR pathway is an important candidate pathway. (C) Heatmap of inflammation-related gene expression (red, high expression; blue, low expression). (D) GO analysis indicating the biological functions of differentially expressed genes, including biological process, molecular function, and cellular component.

Bulk-tissue RNA sequencing analysis was used to identify the “factors of immune regulation” that interacted with HP@CEL through the DNBSEQ-T7 sequence platform. As shown in the volcano plots of all detected and expressed genes (Fig. 8A), a total of 329 genes were identified to be differentially expressed (*P* < 0.05 and |log_2_ fold change| > 2), including 229 down-regulated genes and 100 up-regulated genes. Then, KEGG analysis was performed to reveal the key inflammation signal pathways. Among the top 20 signal pathways, 2 important pathways in red font were related to inflammation (Fig. 8B). KEGG analysis showed that differentially expressed genes were most abundant in leishmaniasis, osteoclast differentiation, tuberculosis, nonobese diabetic (NOD)-like receptor signaling pathway, RA, inflammatory bowel disease (IBD), TLR signaling pathway, and chemokine signaling pathway. The results showed that HP@CEL played an active role in the regulation of the bone immune microenvironment. The first 31 down-regulated and up-regulated differentially expressed genes based on log fold change were shown in the heatmap (Fig. 8C). During the treatment of arthritis in mice, HP@CEL showed a significant downward trend in inflammatory factors compared with the untreated group. Next, the above differentially expressed genes were analyzed by Gene Ontology (GO) enrichment, and most of them were involved in immune regulation, including immune system process, defense response, immune response, plasma membrane part, external side of plasma membrane, integral component of plasma membrane, signaling receptor binding, antigen binding, immunoglobulin receptor binding, and immunoglobulin G binding (Fig. 8D), which also implied the immunomodulation effect of HP@CEL. Detailed information of GO was given in Table S7. These results are consistent with the in vitro results. Bulk-tissue RNA sequencing analysis showed that HP@CEL could alleviate the chronic inflammation of RA and block the progression of RA by down-regulating the level of proinflammatory cytokines.

## Conclusion

Inspired by the unique structure of synovial lipid–HA complex, we successfully prepared a self-assembled polymer hydrogel as joint lubricant to reduce friction and as a local depot to sustained release of CEL for modulating the chronic inflammation for RA treatment. The HP@CEL hydrogel holds merits including thermosensitive property and injectability, self-assemble ability to enable fast in situ formation after injection, sustainable drug release, and well biodegradability. Moreover, we demonstrate that HP@CEL can suppress the LPS-induced inflammatory responses of macrophages and further activate the cross-talk of macrophages and synovial fibroblast cells through inhibiting the TLR4/NF-κB pathways. In the CIA animal models, HP@CEL effectively reduced the local inflammation and improved the bone regeneration through dampening M1 macrophage infiltration and down-regulating the proinflammatory cytokines. Our study highlights the importance of the cross-talk of M1-type macrophages and synovial fibroblast in relieving the positive feedback of the chronic inflammation during the progression of RA. In sum, supramolecular polymer-nanomedicine hydrogel with synovial lipid–HA complex-like structure provides a promising platform for designing immunomodulatory biomaterials toward the treatment of inflammatory diseases including but not limited to RA.

## Ethics Approval

All animal procedures were approved by Animal Experiment Ethics Committee and Authority of Institute of Radiation Medicine, Chinese Academy of Medical Sciences [approval no: SYXK (Jin) 2019-0002].

## Data Availability

All data generated or analyzed during this study are included in this published article or the Supplementary Materials.
