# Supplementary material for: Hyaluronic-Acid-Nanomedicine Hydrogel for Enhanced Treatment of Rheumatoid Arthritis by Mediating Macrophage–Synovial Fibroblast Cross-Talk
Source: Biomater Res. 2024 Jun 18;28:0046. doi: 10.34133/bmr.0046 (PMC11185174; doi:10.34133/bmr.0046)
Supplement: Supplementary 1 — Figs. S1 to S21 Tables S1 to S7 Movies S1 to S6 [file bmr.0046.f1.zip › BMR-D-24-00099R1 SM.docx]

**Hyaluronic acid-nanomedicine hydrogel** **for enhanced treatment of** **rheumatoid arthritis by mediating macrophage-synovial fibroblast crosstalk**

Yaping Wang^1^, Jingrong Wang^2^, Mengze Ma^1^, Rui Gao^2^, Yan Wu^1^, Chuangnian Zhang^2^, Pingsheng Huang^2^, Weiwei Wang ^2, 3*^, Zujian Feng ^2*^ and Jianbo Gao ^1*^

^1^ Medical 3D Printing Center, The First Affiliated Hospital of Zhengzhou University, Zhengzhou 450000, China. ^2^ Tianjin Key Laboratory of Biomaterial Research, Institute of Biomedical Engineering, Chinese Academy of Medical Sciences and Peking Union Medical College, Tianjin 300192, China. ^3^ Key Laboratory of Innovative Cardiovascular Devices, Chinese Academy of Medical Sciences, Beijing 100144, China

* Address corresponding to: wwwangtj@163.com (W. Wang); fzujian@163.com (Z. Feng); cjr.gaojianbo@vip.163.com (J. Gao)

**SUPPLEMENTARY MATERIALS AND METHODS**

**Materials**

PEG (average Mn=1500 g/moL), ε-caprolactone (CL, 97%), and stannous octoate were obtained from Shanghai Aladdin Bio-Chem Technology Co. Ltd. (Shanghai, People’s Republic of China). Tetrahydrofuran (THF) and dimethyl sulfoxide were purchased from Concord Co. Ltd. (Tianjin, People’s Republic of China). All chemical reagents were used without further purification. Triton X-100, LPS, DAPI and Actin-Tracker Red-555 were purchased from Beyotime Biotechnology (Shanghai, China). Celastrol (CEL), Alizarin Red, Sirius Red staining and Rhodamine B were purchased from Sigma-Aldrich. Dulbecco’s modified Eagle’s medium, fetal bovine serum, and penicillin-streptomycin. Fluorescent dye-labeled monoclonal antibodies (CD68, CD86 and F4/80) were provided by eBioscience (San Diego, California, USA). Primary antibodies to CD68, CD86, TLR4, MyD88, MAP3K7(p38), NF-κB(p65), IκBα, β-Actin and secondary antibodies were purchased from Abcam (Cambridge, UK). All other chemical agents and solvents were analytical grade.

**Experimental method**

*Molecular dynamics simulation of PECT nanoparticles:* LAMMPS 2022 software was used to simulate the self-assembly behavior of coarse-grained PECT copolymers in water environment, where yellow spheres represent coarse-grained PEG monomers, blue and red represent CL and TOSUO monomers, respectively.

*Preparation of 5-ethylene glycol ketal-ε-caprolactone (TOSUO):* The preparation route of TOSUO is shown in Figure S4. 1,4-cyclohexanedione monoethylene ketal was added to a single bottle, and dichloromethane was added to dissolve it. Then m-chloroperoxybenzoic acid was added in batches. After the reaction was completed, the dichloromethane was dried by filtration and recrystallized three times in ether to obtain pure TOSUO.

*Synthesis of poly(ε-caprolactone-co-1,4,8-trioxa[4.6]spiro-9-undecanone)-poly(ethylene glycol)-poly(ε-caprolactone-co-1,4,8-trioxa[4.6]spiro-9-undecanone) (PECT)*: The triblock copolymer PECT was prepared by ring-opening polymerization using polyethylene glycol as initiator, caprolactone and 5-ethylene glycol ketal-ε-caprolactone as monomers, stannous octoate as catalyst. The specific experimental process is as follows: PEG (1 g, 0.67 mmol), CL (2.51 g, 21.9 mmol) and TOSUO (0.43 g, 2.5 mmol) were added to a 250 mL three-necked bottle, stannous octoate (161 μL, 0.50 mmol) was added after water removal, and the reaction was performed at 130 °C for 12 hours. After the reaction, dichloromethane (5 mL) was added to dissolve the crude product, and then slowly added to the cold ether at -20 °C. The precipitated product was filtered and dried under vacuum to obtain the final product PECT.

*Particle size and temperature sensitivity test of PECT nanoparticles:* The particle size of PECT nanoparticles was characterized by dynamic light scattering (DLS, Brookhaven BI-200SM). The test temperature was 15-60 °C, the scattering angle was 90 °, and the scanning wavelength was 532 nm.

*Synthesis of* *poly(ε-caprolactone-co-1,4,8-trioxa [4.6]spiro-9-undecanone)) (PCT):* The copolymer PCT was prepared by using benzyl alcohol as initiator, caprolactone and 1,4,8-trioxa[4.6]spiro-9-undecanone as monomers, stannous octoate as catalyst. The specific experimental process is as follows: BNOH (346.15 µL, 3.34 mmol), CL (12.2 mL, 114.43 mmol) and TOSUO (2.14 g, 12.43 mmol) were added to a 250 mL three-port bottle, stannous octoate (161 μL, 0.50 mmol) was added after water removal, and the reaction was carried out at 130 °C for 12 hours. After the reaction, dichloromethane (5 mL) was added to dissolve the crude product, and then slowly added to the cold ether at -20 °C. The precipitated product was filtered and dried under vacuum to obtain the final product PCT.

*Synthesis of* *HA-PCT:* The copolymer HA-PCT was prepared by using dicyclohexylcarbodiimide (DCC) as condensation agent and 4-dimethylaminopyridine (DMAP) as catalyst. The specific experimental process was as follows: DMSO (100 mL), HA (1 g, 2.64 mmol), PCT (4 g, 0.528 mmol), DCC (1 g, 4.85 mmol), DMAP (0.064 g, 0.52 mmol) were added to the beaker and reacted for 2 days at room temperature. After the reaction, DMSO and water were used for dialysis. The supernatant was centrifuged and freeze-dried to obtain the final product HA-PCT. The degree of substitution of PCT was determined by nuclear magnetic resonance internal standard method. 10 mg HA-PCT was dissolved in 500 μL DMSO, and 5 μL internal standard acetonitrile was added.

*Synthesis and characterization of PECT and cel-loaded PECT micelle (P@CEL):* PECT (100 mg) was dissolved in tetrahydrofuran (2 mL) and slowly added to double distilled water. After tetrahydrofuran was completely volatilized, the polymer segments self-assembled into nanoparticles with nucleation-shell structure in water. P@CEL was prepared as follows: 100 mg of PECT and 5 mg of CEL were dissolved and mixed in THF (2 mL). Then, the above mixture was slowly added to 10 mL of ddH_2_O. Next, the solvent was completely removed by magnetic stirring for 6 h. The micelles were obtained by centrifugation, supernatant and freeze-drying. ^1^H NMR spectra of the products were recorded on a 400 MHz spectrometer with D_2_O or CDCl_3_ as the solvent depending on the solubility. Dynamic laser scattering measurements were performed on a Zeta Sizer Nano 90 (Malvern Instrument, UK). TEM (JEM-1011; JEOL, Tokyo, Japan) was used to characterize the morphology of the micelle. All measurements were in triplicate. The drug loading efficiency and encapsulation efficiency were calculated based on the following equations.

$$\text{Drug loading efficiency}\text{ (\%)=}\frac{\text{Amount of loaded drug}}{\text{Total amount of drug (dosage)}}\text{×100\%}$$

$$\text{Encapsulation efficiency (\%)=}\frac{\text{Encapsulation efficiency}}{\text{Theoretical loading}}\text{×100\%}$$

*Synthesis and characterization of* *PECT and P@CEL hydrogel (**P@CEL)*: PECT (30 mg) or P@CEL (30 mg) was dissolved in deionized water (100 µL) and stirred well at 25 °C. Hydrogel was formed at 37 °C.

*The release of CEL from the* *hydrogel in vitro:* HP@CEL was immersed in PBS supplemented with 0.5 U/mL hyaluronidase to simulate the release of drug in vivo. The HP@CEL was then incubated at 37 °C. The supernatant was collected and replenished with fresh solution at scheduled time point (7^th^, 14^th^, 21^th^, 28^th^, 35^th^, 42^th^, 49^th^, 56^th^, 63^th^, 70^th^ day). The collected solution was determined by the UV–vis spectrophotometer, and the drug release percentage (%) were calculated based on the following equations.

$$\text{Drug release (\%)=}\frac{\text{Amount of released drug}}{\text{Total amount of loaded drug}}\text{×100\%}$$

*Rhodamine B fluorescent labeling and in vivo imaging after subcutaneous implantation:* The degradation of HP@CEL *in vivo* was investigated by fluorescence imaging to visualize the gel in a real-time manner. PECT (2.332 g, 0.4 mmol), DCC (600 mg), DMAP (354 mg) and rhodamine B (460 mg) were placed in a 500 mL single-necked bottle and dissolved in 200 mL anhydrous dichloromethane for 24 h. After the end of the reaction, dialysis with anhydrous ethanol for 72 hours. Then PECT-RB was obtained by precipitation, filtration and drying in cold ether. the PECT was labeled with rhodamine B (RB). RB-P@CEL was then mixed with HA-PCT to form hydrogel. Subsequently, HP@CEL (200 µL) was injected subcutaneously at the back of female BALB/c mice (n = 3). The fluorescence signal was detected by the CRI Maestro imaging system (CRI Corporation, Woburn, MA, USA) and quantified using region of interest (ROI) analysis. The total fluorescence signal (TFS) was expressed as mean ± SD (n = 3) at different time points.

*Polarization of macrophages in vitro:* RAW264.7 were seeded in a 6 well plate at the density of 4 × 105 cells per well, and pre-treated with LPS (40 ng mL^−1^) to obtain the M1 type macrophages. Then, PBS, CEL, HA-PCT, PECT or HP@CEL in fresh culture medium was added and incubated for further 48 h. Cells were collected by centrifugation and washed twice with cold PBS. Collected cells were stained with FITC-labeled anti-CD86 antibodies, PE-labeled F4/80 antibodies and analyzed by flow cytometry.

*Macrophage and synovial cell co-culture:* To study the effect of M1-proinflammatory factor on synovial fibroblasts, M1 type macrophages were co-cultured with synovial fibroblasts. M1 type macrophages were obtained through pre-treated with LPS as described above, and further incubated with PBS, CEL, HA-PCT, PECT or HP@CEL in fresh culture medium for further 48 h. Then, the supernatants of treated macrophages were collected to stimulate synovial fibroblasts for 24 h. Total RNA of the treated synovial fibroblasts was extracted, and the expression level of TLR4, MyD88, MAP3K7(p38), NF-κB(p65), TNFα, IL-1β, IL-6 was measured by the RT-qPCR.

*3D fluorescent images of the* *BMSCs cells cultured on the hydrogel for 24 h*: BMSCs were isolated from SD rats (male, 6 weeks) following standard procedures. BMSCs were treated with CEL, P@CEL or HP@CEL (the equivalent of 100 ng/mL of CEL) for 24 h. BMSCs solution (1 × 10^7^ cells/mL) was incubated with P@CEL and HP@CEL hydrogel. Cells were fixed and stained with DAPI and Actin-Tracker Red-555 in the dark. The fluorescence images were then obtained using a laser scanning confocal microscope.

*Cell proliferation was further evaluated by cell counting kit-8 assay kit (CCK-8):* To estimate the biocompatibility of HP@CEL, cell viability was detected using CCK-8 assay. Briefly, cells after treatment were rinsed with PBS and incubated for 3 h with sterilized 10% CCK-8 solution. The absorbance at 450 nm was measured with a microplate reader (Thermo Scientific Appliskan, USA).

*Cell staining and confocal imaging:* The BMSCs after HP@CEL hydrogel treatment were fixed with 4% paraformaldehyde and permeabilized with a 0.1% Triton X-100 solution in PBS. Subsequently, the BMSCs were stained with Actin-Tracker Red-555 (dilution 1:200) for 40 min and DAPI for 5 min. Samples were rinsed three times with sterile PBS. Images were captured by CLSM and quantitatively analyzed by Image J software.

*Staining and quantification of ECM mineralization and collagen secretion*: Mineralized extracellular matrix and collagen secretion were evaluated with the use of Alizarin Red S and Sirius Red staining, respectively. At 14 days post incubation, the cells or cell-encapsulated HP@CEL were immersed in 4% paraformaldehyde for 5 min and rinsed three times with PBS. The fixed cells were incubated in 1% Alizarin red S solution or Sirius Red solution for 5 min, respectively. The stained cells were observed. After carefully washed with deionized water, the stained cells were dried and photographed. The absorbance at a wavelength of 540 nm or 620 nm was measured by a microplate reader.

*Real-time quantitative reverse transcription polymerase chain reaction (RT-qPCR)*: Total RNA was extracted using the HR Total RNA kit (OMEGA, R6812-02), and one microgram of RNA was used to synthesize cDNA using a reverse transcription reagent kit. Then, the cytokines including TLR4, MyD88, MAP3K7(p38), NF-κB(p65), TNFα, IL-1β, IL-6 was measured by the RT-qPCR based on SYBR Green Master Mix (Promega, A6001). PikoReal 96 Real-time Thermal Cycler (Thermo Fisher Scientific, Finland) was used for RT-qPCR analysis using Real Master Mix (SYBR Green) (NEWBIO, China) and a thermal cycling was performed at 95 °C for 2 min, followed by 45 cycles at 95 °C for 20 s and 58 °C for 20 s, 72 °C for 30 s.

In order to study the role of hydrogels in the treatment of arthritis, the expression of specific genes in articular bone was quantitatively determined. The hind paws of anesthetized mice were collected on day 50 (30 days after treatment). After removing the skin and muscle, 600 µL TRIZOL was added to grind the joint bone into homogenate. The above homogenate samples were transferred to a 1.5 mL EP tube and placed at 15-30 °C for 5 minutes to completely separate the nucleoprotein complex. Add 0.2 mL of chloroform, vortex shake for 15 seconds, place at 15-30 °C for 2-3 minutes, and centrifuge. The upper aqueous phase was added with 0.5 mL isopropanol, allowed to stand at room temperature for 10 minutes, and then centrifuged to obtain RNA. Aggrecan was used as the reference gene and the primer sequences for TLR4, MyD88, MAP3K7(p38), NF-κB (p65), TNF-α, IL-1β, and IL-6 were listed in Table S6.

*Western Blot:* RIPA lysis buffer (Beyotime, P0013B) was applied to obtain total cell lysates. Primary antibodies against TLR4 (CST, 14358S), MyD88 (Abcam, ab219413), MAP3K7(p38) (Abcam, ab228752), NF-κB(p65) (Abcam, ab16502) and IκBα (Abcam, ab32518) were used for protein staining according to the manufacturer’s recommendations. Proteins were extracted, dissolved by SDS-PAGE and then transferred to 0.22 μm polyvinylidene difluoride (PVDF) membranes. The PVDF membrane was blocked with 5 wt% BSA in TBST for 2 h at room temperature and probed with the indicated primary antibodies at 4 ℃ overnight, which was rinsed and incubated with dilutions of appropriate secondary antibodies conjugated with horseradish peroxidase (Cell Signaling Technology) for 1 h at room temperature followed by incubation with an enhanced chemiluminescence kit (Bio-Rad, USA) for a few seconds. Proteins on the membranes were visualized by Chemiluminescent HPR Substrate and images were captured by Chemiluminescence Imaging system (ChemiScope 6000 Pro, China). The signal intensity of immunoreactive bands was quantified by Image J software and normalized using β-actin.

*Photograph:* In order to evaluate of joint swelling, the hind paws of mice were measured at day 20, 23, 26, 29, 32, 35, 38, 41, 44, 47, and 50 after treatment by a camera.

*Arthritis induction and treatment:* All mice were stabilized for at least 1 week. To induce arthritis, 100 μg of chicken type II collagen (CII, Chondrex Inc., Redmond, USA) and 100 μL complete Freund’s adjuvant (CFA, Sigma F5881) were completely emulsified and injected into the paw of the right hind limb, followed by a boosting immunization on day 14 using another 100 μg of CII. On day 20, collagen-induced arthritis (CIA) mice were divided into five groups randomly, including CIA control group, CEL, HA-PCT, PECT and HP@CEL treated group, respectively. CEL treated groups were then administrated via tail vein at a dose of 10 mg/kg at day 20, 30 and 40, respectively. HA-PCT, PECT and HP@CEL (50 µL) was injected subcutaneously at the paw of the right hind limb. The positive control group of mice received only an equal volume of saline by the same method and at the same days. Each group contained eight mice. The mice were reared alone after surgery and euthanized by CO_2_ asphyxiation 50 days later.

*Clinical score and measurement:* After the start of therapy, mice were observed and weighed every 3 days. Grading according to the severity of edema and deformation of each paw joint in mice. The severity of joint pathology was graded according to whether there was edema and deformation in the joint, and the degree of each claw was 0-4. the maximum arthritic score per mouse was obtained by adding the score of individual paws so that the maximum possible score was 16. The claw thickness of the ankle joint of both hind feet was measured by vernier caliper scoring method.

*Histopathologic evaluation:* After the sacrifice of the mice at day 50, hind legs were removed from CIA mice. Legs were embedded and sliced after decalcification. The sections were stained with hematoxylin and eosin (H & E), toluidine blue (T & B) and safranine O-fast green FCF cartilage staining (SO-FG) to assess inflammation and joint destruction.

*Immunofluorescent staining:* The sections of the samples were rinsed with PBS for 3 times, and then blocked with goat serum. The sections were incubated with primary antibody at 4 ℃ overnight. Next, the slices were incubated with the corresponding secondary antibody. Finally, the slices were stained with DAPI, the images were taken and quantitative analysis was performed.

**Supplementary Tables and Figures**

**Table S1**. 24 potential target genes of Celastrol.

| No. | Target | Gene name |
| --- | --- | --- |
| 1  2  3  4  5  6  7  8  9  10  11  12  13  14  15  16  17  18  19  20  21  22  23  24 | Vascular endothelial growth factor A  G1/S-specific cyclin-D1  Apoptosis regulator Bcl-2  Bcl-2-like protein 1  Apoptosis regulator BAX  Vascular endothelial growth factor receptor 1  Vascular endothelial growth factor receptor 2  Matrix metalloproteinase-9  Transcription factor AP-1  Prostaglandin G/H synthase 2  Baculoviral IAP repeat-containing protein 5  Baculoviral IAP repeat-containing protein 2  CASP8 and FADD-like apoptosis regulator  Aldose reductase  Hsp90 co-chaperone Cdc37  72kDa type IV collagenase  Metalloproteinase inhibitor 1  Metalloproteinase inhibitor 2  Interstitial collagenase  Myc proto-oncogene protein  Fibronectin  Collagen alpha-1(VII) chain  Transforming growth factor beta-1  Collagen alpha-4(IV) chain | VEGFA  CCND1  BCL2  BCL2L1  BAX  FLT1  KDR  MMP9  JUN  PTGS2  BIRC5  BIRC2  CFLAR  AKR1B10  CDC37  MMP2  TIMP1  TIMP2  MMP1  MYC  FN1  COL7A1  TGF-β1  Col4A4 |

**Table S2**. 9 potential target genes of Celastrol therapy for RA.

| No. | Target | Gene name |
| --- | --- | --- |
| 1  2  3  4  5  6  7  8  9 | Vascular endothelial growth factor A  Matrix metalloproteinase-9  Transcription factor AP-1  Prostaglandin G/H synthase 2  72kDa type IV collagenase  Metalloproteinase inhibitor 1  Metalloproteinase inhibitor 2  Interstitial collagenase  Transforming growth factor beta-1 | VEGFA  MMP9  JUN  PTGS2  MMP2  TIMP1  TIMP2  MMP1  TGF-β1 |


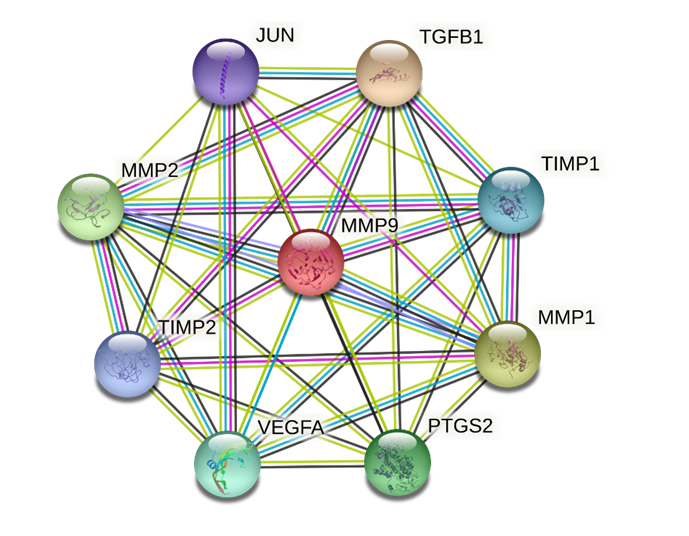


Figure S1 PPI network map.


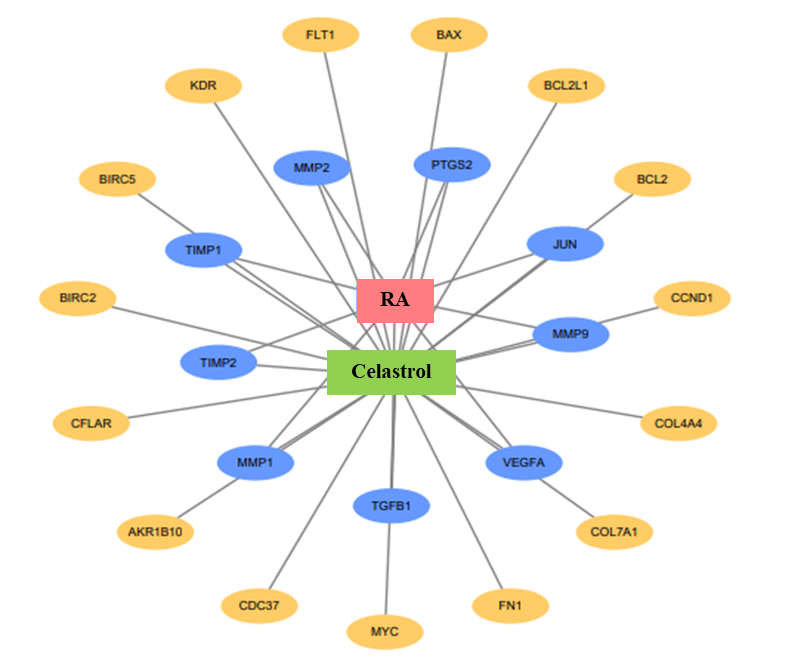


Figure S2 The celastrol–RA-potential target gene network.


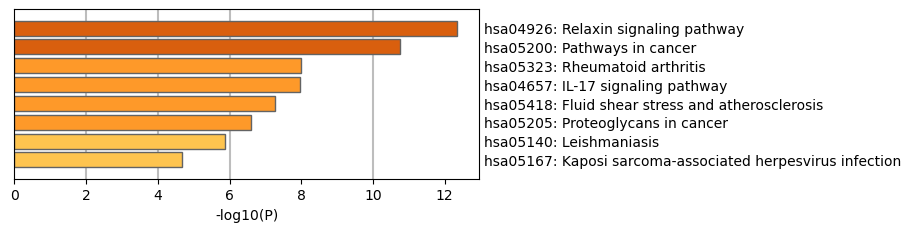
Figure S3 KEGG pathway analysis of 24 potential target genes of celastrol.

Figure S4 The synthesis of TOSUO.

Figure S5 The synthesis of PECT.

Figure S6 The synthesis of PCT.


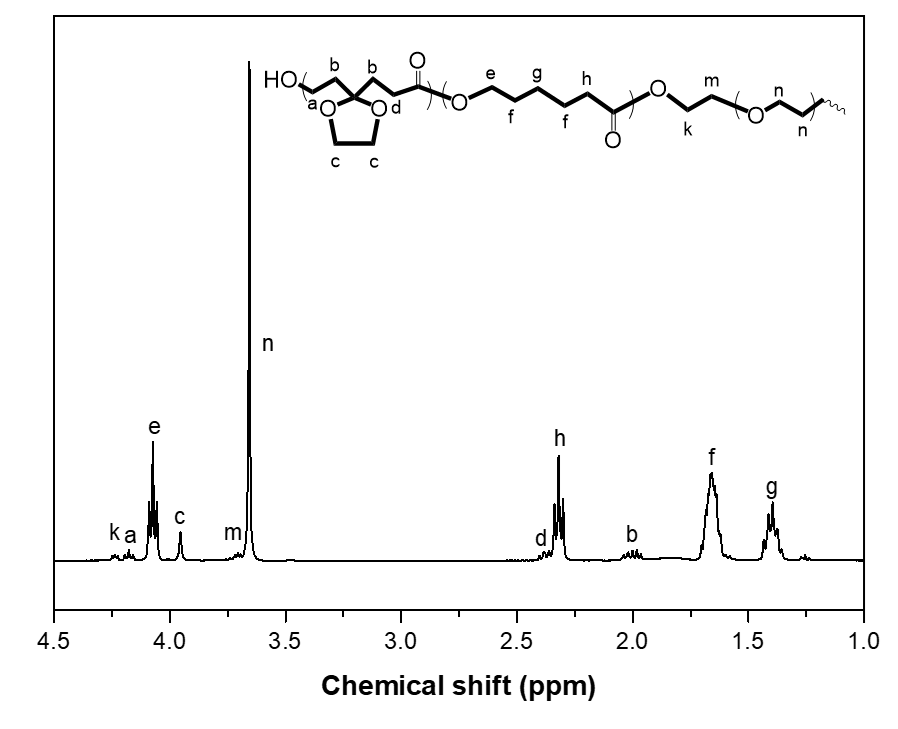


Figure S7 ^1^H NMR spectrum of PECT.


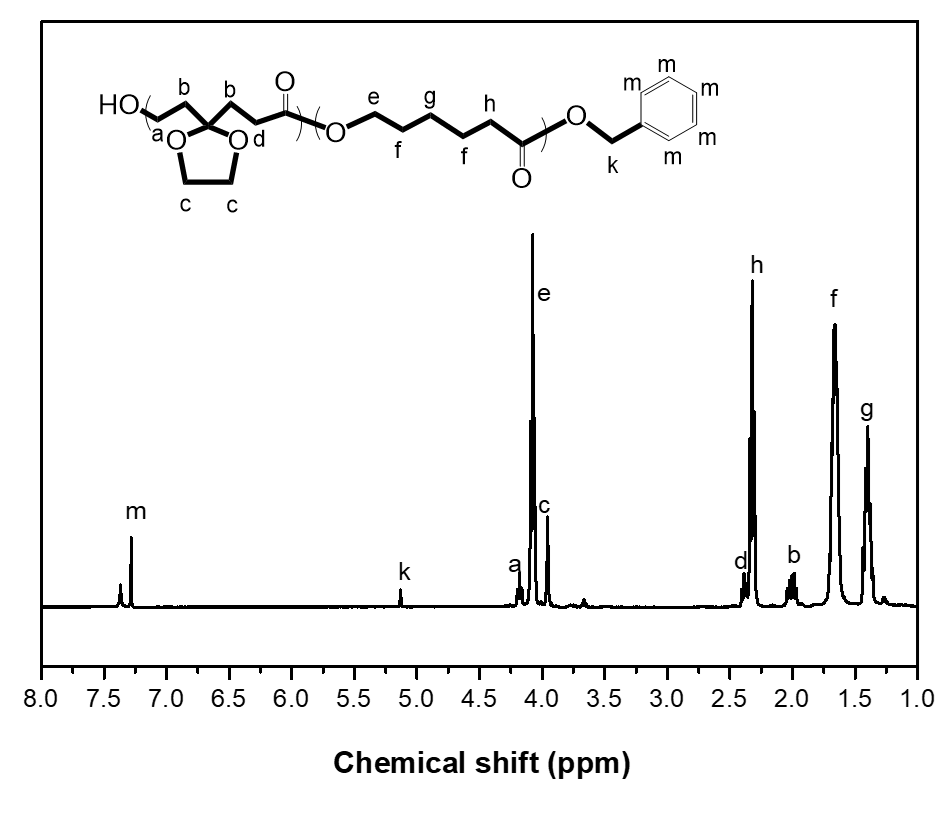


Figure S8 ^1^H NMR spectrum of PCT.

Figure S9 The GPC spectrum of PECT、PCT and HA-PCT.


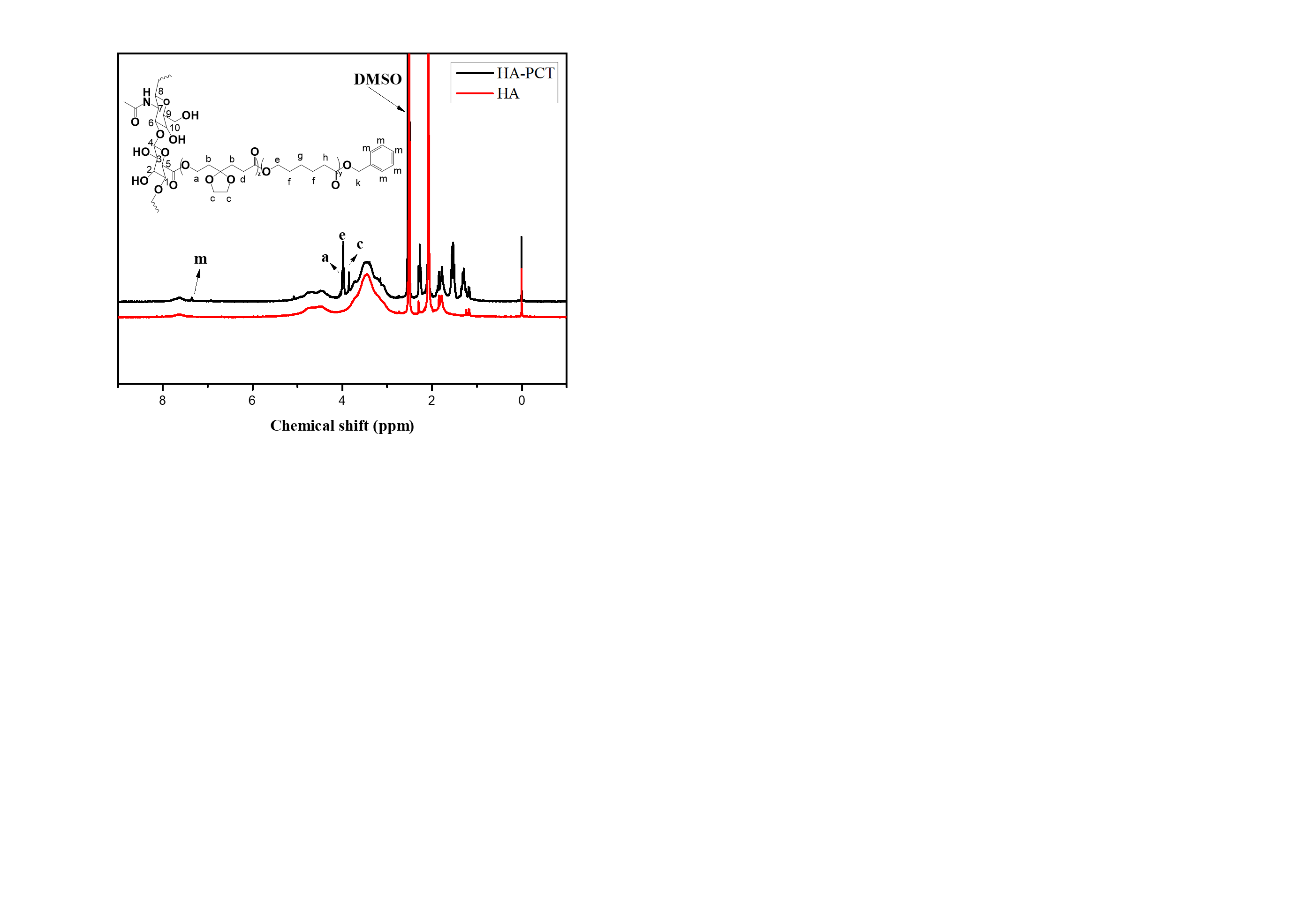


Figure S10 ^1^H NMR spectrum of HA-PCT.

Table S3. Composition of PECT.

| Theoretical  composition | EG: CL: TOSUO | | M^a^ | M_n_^b^ | M_p_^b^ | PDI^b^ |
| --- | --- | --- | --- | --- | --- | --- |
|  | Feed ratio | Product^a^ |  |  |  |  |
| PECT | 34:33:3.75 | 34:38:6 | 6860 | 3879 | 6728 | 1.84 |

^a^ The polymerization degree of each monomer were calculated from ^1^H-NMR.

^b^ Polymer molecular weight and distribution were determined by GPC.

Table S4. Composition of PCT and HA-PCT.

| Theoretical  composition | BNOH: CL: TOSUO | | M^a^ | M_n_^b^ | M_p_^b^ | PDI^b^ |
| --- | --- | --- | --- | --- | --- | --- |
|  | Feed ratio | Product^a^ |  |  |  |  |
| PCT | 1:41.63:3.78 | 1:57:5.6 | 5472.98 | 4302 | 7669 | 2.00 |
| HA-PCT |  |  |  | 3084 | 8193 | 2.67 |

^a^ The polymerization degree of each monomer were calculated from ^1^H-NMR.

^b^ Polymer molecular weight and distribution were determined by GPC.

Figure S11 The FI-TR spectrum of HA, PCT and HA-PCT.


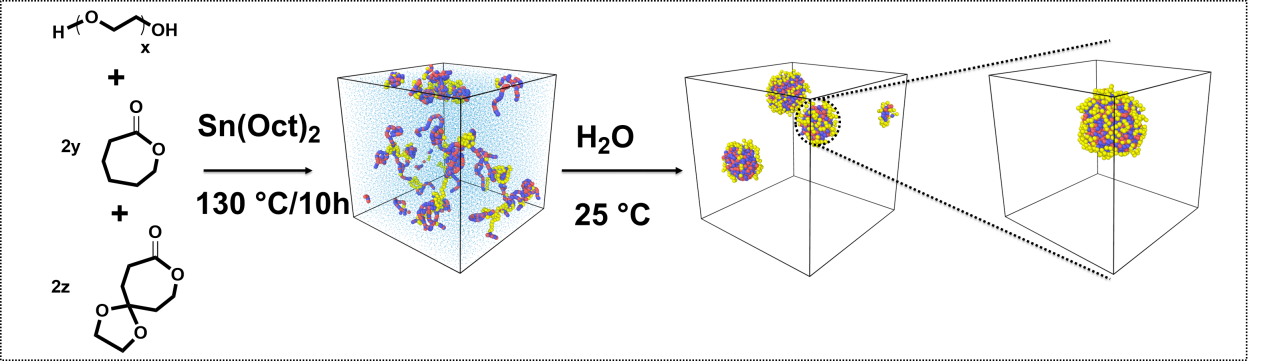
Figure S12 MD modeling of formation of PECT nano micelles.

Table S5. Size, PDI, drug loading efficiency (DLE) and encapsulation efficiency (DEE) of PECT and P@CEL nanoparticles.

|  | Size (nm)^a^ | PDI^a^ | DLE | DEE |
| --- | --- | --- | --- | --- |
| PECT | 126 | 0.24 | / | / |
| P@CEL | 146 | 0.24 | 2% | 40% |


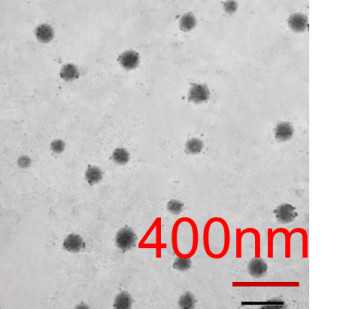


Figure S13 TEM images of nanoparticles in HP@CEL hydrogel degradation solution.


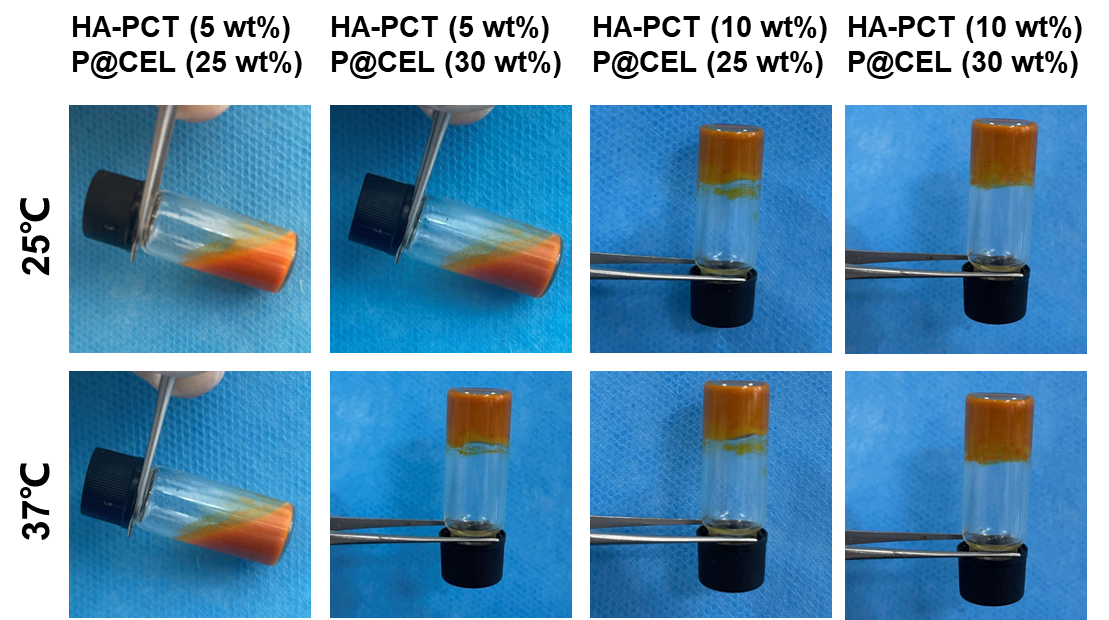


Figure S14 The photos of the sol–gel transition of HP@CEL hydrogel at 37℃.


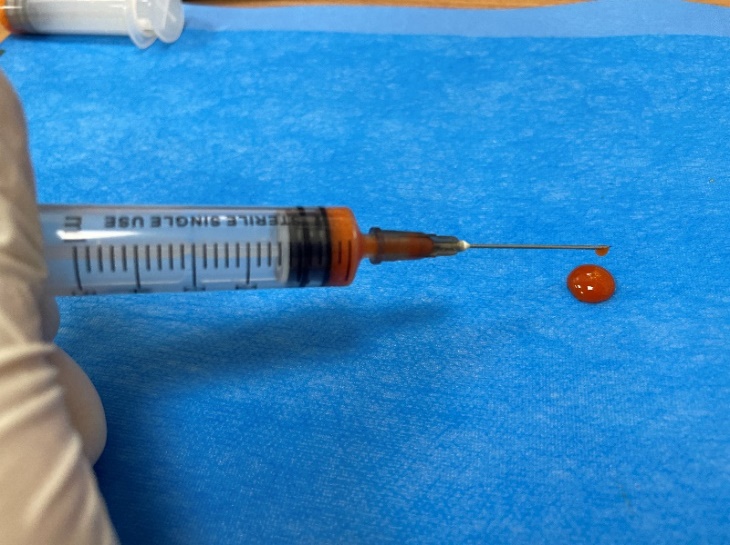


Figure S15 Photographic image of injectable HP@CEL hydrogel.

Figure S16 The degradation of HP@CEL hydrogel at 37 °C.

Figure S17 The cumulative release profile of CEL from the HP@CEL and P@CEL hydrogel in vitro.


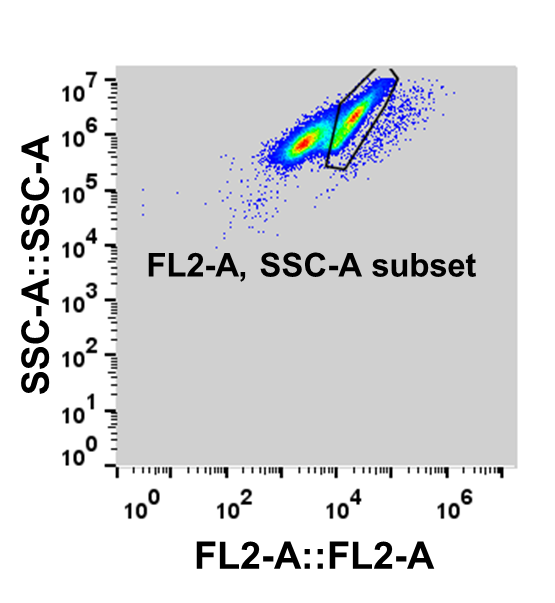


Figure S18 The gating approach for flow cytometry.


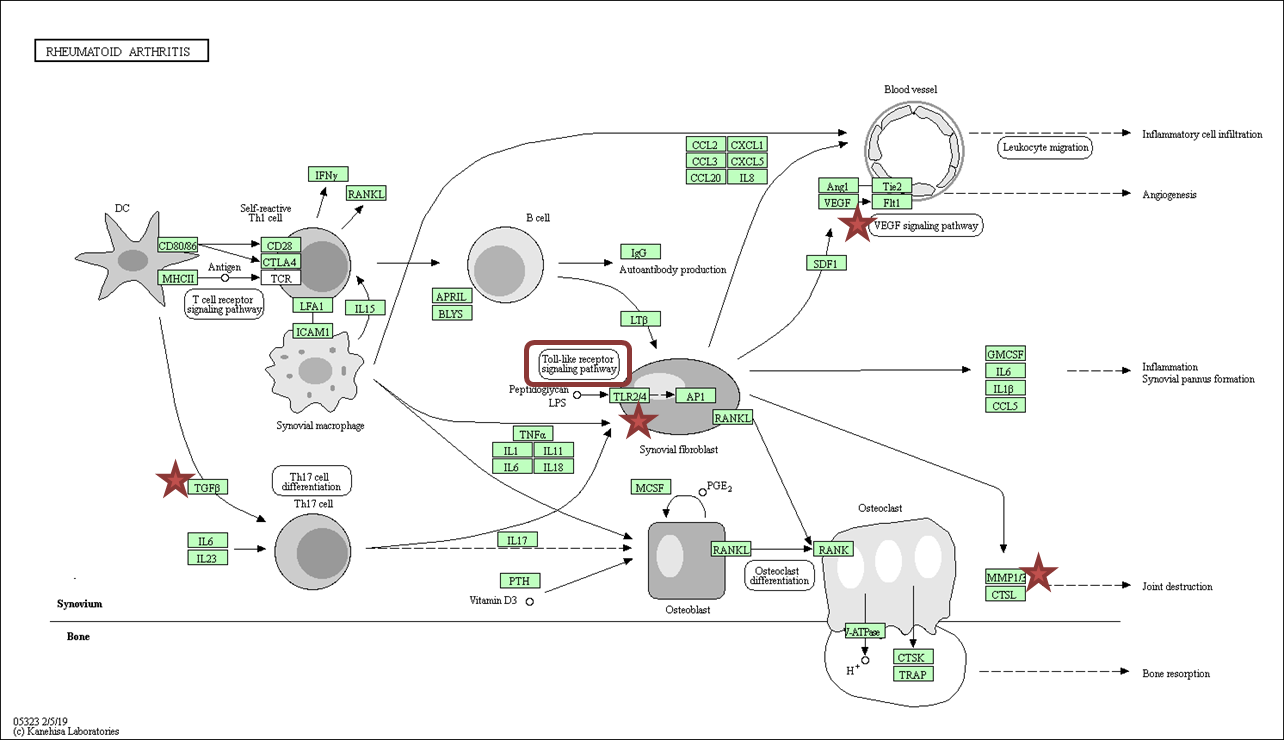


Figure S19 The rheumatoid arthritis pathway of celastrol.


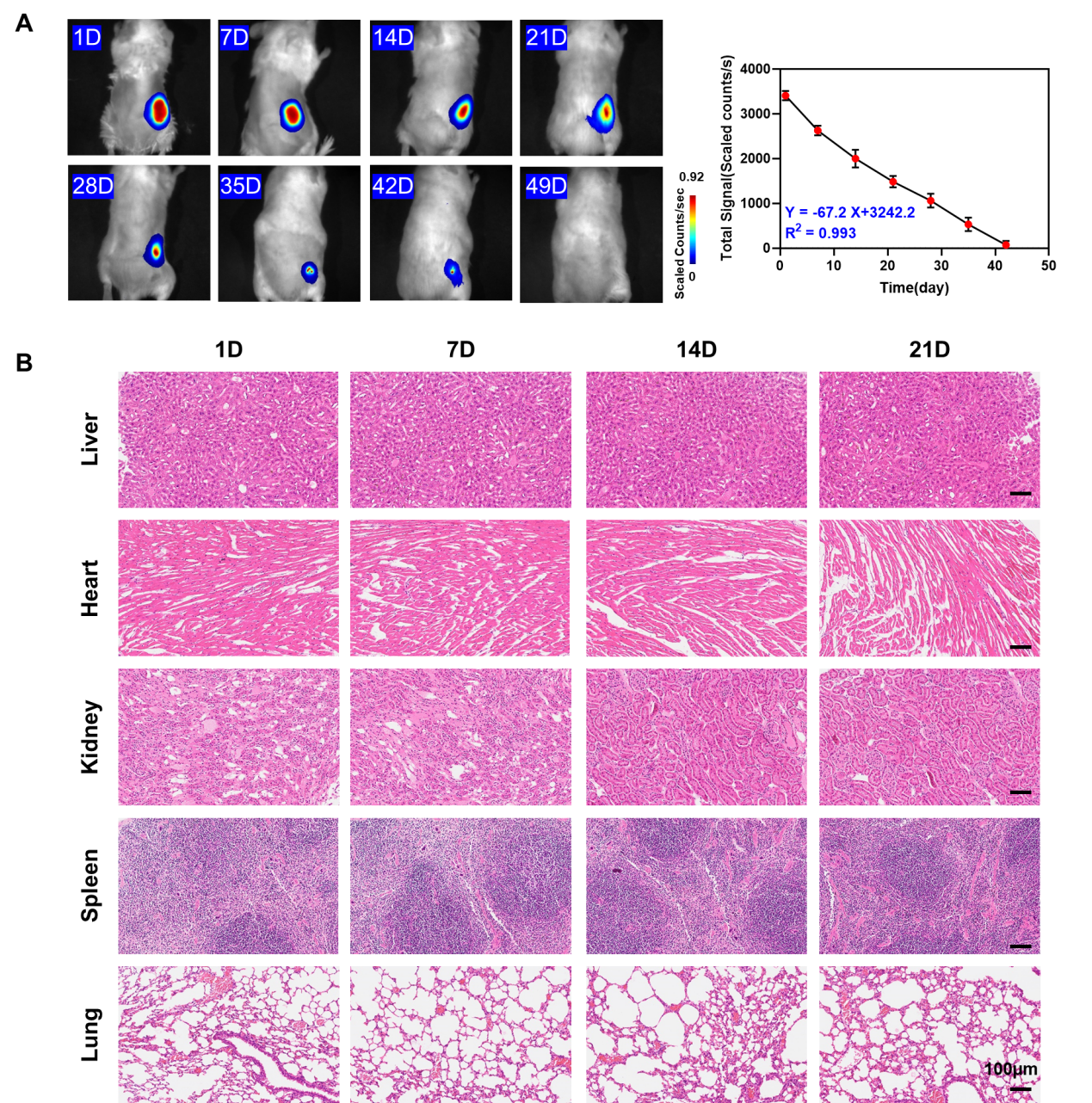


Figure S20 (A) Time dependent fluorescent images of mice received subcutaneous injection with RB-labeled HP@CEL hydrogel and the fluorescence intensity of RB at different time points after injection. (B) Representative H&E staining images of liver, heart, spleen, lung, kidney and skin.


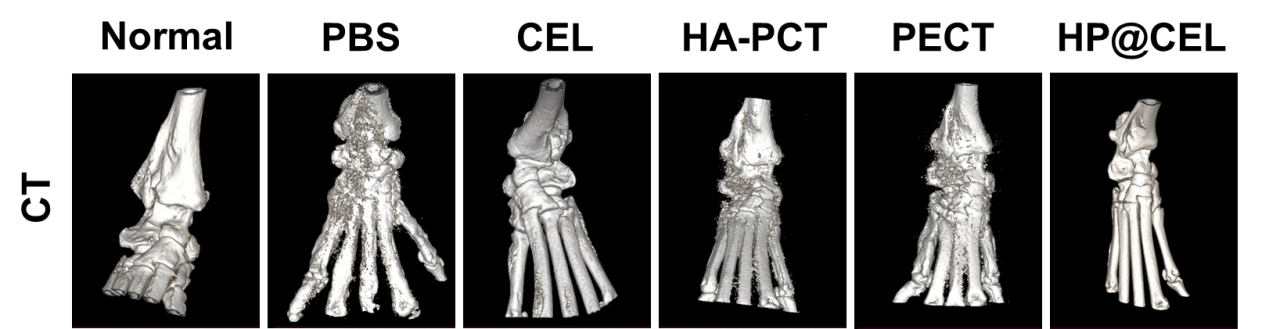


Figure S21 Micro-CT images.

Table S6 Sequences of primers used in the RT-qPCR.

| Gene | Forward primer (5’-3’) | Reverse primer (5’-3’) |
| --- | --- | --- |
| TLR4 | TGGTTGCAGAAAATGCCAGG | TCATCAGGGACTTTGCTGAGTT |
| MyD88 | GCTGTTCTTGAACCCTCGGA | GCCAGGCATCCAACAAACTG |
| NF-κB | GCCTCTGGCGAATGGCTTTA | TGCTTCGGCTGTTCGATGAT |
| MAP3K7  IκBα  TNFα  IL-1β  IL-6 | CCTCGTCTTCTGCCAGTGAG  TGACTTTGGGTGCTGATGTC  CCCTCACACTCACAAACCAC  TGCCACCTTTTGACAGTGATG  GTCCTTCCTACCCCAATTTCCA | GCTCCTCTTCCGACAACCTC  AAGCTGGTAGGGGGAGTAGC  ACAAGGTACAACCCATCGGC TGATGTGCTGCTGCGAGATT  TAACGCACTAGGTTTGCCGA |

Table S7. The name of the pathway represented by the ID of GO.

| ID | Name |
| --- | --- |
| GO:0065007  GO:0050789  GO:0050794  GO:0050896  GO:0048518  GO:0048522  GO:0002376  GO:0048583  GO:0032502  GO:0048519  GO:0005515  GO:0005102  GO:0042802  GO:0044877  GO:0003823  GO:0005539  GO:0034987  GO:0030246  GO:0005126  GO:0001664  GO:0044425  GO:0016020  GO:0031224  GO:0016021  GO:0005886  GO:0044459  GO:0044421  GO:0031226  GO:0005887  GO:0005615 | biological regulation  regulation of biological process  regulation of cellular process  response to stimulus  positive regulation of biological process  positive regulation of cellular process  immune system process  regulation of response to stimulus  developmental process  negative regulation of biological process  protein binding  signaling receptor binding  identical protein binding  protein-containing complex binding  antigen binding  glycosaminoglycan binding  immunoglobulin receptor binding  carbohydrate binding  cytokine receptor binding  G protein-coupled receptor binding  membrane part  membrane  intrinsic component of membrane  integral component of membrane  plasma membrane  plasma membrane part  extracellular region part  intrinsic component of plasma membrane  integral component of plasma membrane  extracellular space |
